# Genome wide association study of 40 clinical measurements in eight dog breeds

**DOI:** 10.1038/s41598-020-63457-y

**Published:** 2020-04-16

**Authors:** Yukihide Momozawa, Anne-Christine Merveille, Géraldine Battaille, Maria Wiberg, Jørgen Koch, Jakob Lundgren Willesen, Helle Friis Proschowsky, Vassiliki Gouni, Valérie Chetboul, Laurent Tiret, Merete Fredholm, Eija H. Seppälä, Hannes Lohi, Michel Georges, Anne-Sophie Lequarré

**Affiliations:** 1 Unit of Animal Genomics, GIGA Institute, University of Liège, Liège, Belgium; 2 Laboratory for Genotyping Development, RIKEN Center for Integrative Medical Sciences, Kanagawa, 230-0045 Japan; 3 Department of Clinical Sciences, Faculty of Veterinary Medicine, University of Liège, Liège, Belgium; 4 Department of Equine and Small Animal Medicine, Faculty of Veterinary Medicine, University of Helsinki, Helsinki, Finland; 5 Faculty of Health and Medical Sciences, University of Copenhagen, 1870 Frederiksberg C, Denmark; 6 The Danish Kennel Club, 2680 Solrød Strand, Denmark; 7 U955 - IMRB Inserm and Unité de Cardiologie d’Alfort (UCA), Université Paris-Est, École Nationale Vétérinaire d’Alfort, UPEC, 7 avenue du général de Gaulle, Maisons-Alfort, F-94700, France; 8 U955 - IMRB, Biology of the neuromuscular system, Inserm, National Veterinary School of Alfort (ENVA), Maisons-Alfort, France; 9 Department of Veterinary Biosciences, Department of Medical and Clinical Genetics, University of Helsinki, Folkhälsan Research Center, Helsinki, Finland

**Keywords:** Genome-wide association studies, Quantitative trait loci, Animal breeding

## Abstract

The domestic dog represents an ideal model for identifying susceptibility genes, many of which are shared with humans. In this study, we investigated the genetic contribution to individual differences in 40 clinically important measurements by a genome-wide association study (GWAS) in a multinational cohort of 472 healthy dogs from eight breeds. Meta-analysis using the binary effects model after breed-specific GWAS, identified 13 genome-wide significant associations, three of them showed experimental-wide significant associations. We detected a signal at chromosome 13 for the serum concentration of alanine aminotransferase (ALT) in which we detected four breed-specific signals. A large proportion of the variance of ALT (18.1–47.7%) was explained by this locus. Similarly, a single SNP was also responsible for a large proportion of the variance (6.8–78.4%) for other measurements such as fructosamine, stress during physical exam, glucose, and morphometric measurements. The genetic contribution of single variant was much larger than in humans. These findings illustrate the importance of performing meta-analysis after breed-specific GWAS to reveal the genetic contribution to individual differences in clinically important measurements, which would lead to improvement of veterinary medicine.

## Introduction

The domestic dogs share many diseases and phenotypes with human^1^. Through two bottlenecks resulting from domestication^2^ and the frequent use of specific males, each dog breed shows lower heterogeneity for disease. In humans, higher heterogeneity, meaning multiple variants with different levels of effects influencing the same disease, makes it difficult to identify genetic variants associated with disease^3^. Therefore, dogs are considered an ideal model animal for identifying genes and genomic loci underlying diseases and phenotypic variation^1,4^. Genome-wide association studies (GWAS) using canine single nucleotide polymorphism (SNP) chips have identified susceptibility genes and genomic loci for several complex diseases^5–10^ and for many Mendelian disorders^11–16^.

The diagnosis of a disease often involves taking many clinical measurements including blood and urine analysis. These measurements directly reflect different aspects of the health of each individual. Individual differences in these measurements are known to be heritable in humans, and GWAS have identified genetic loci explaining these individual differences^17^. A systematic analysis of 6,046 dogs has identified breed-specific differences in hematological measurements^18^, suggesting also a genetic contribution to the inter-breed variation for these measurements. Identification of the responsible genes would lead to a better understanding of the biological mechanism under the intermediate phenotypes for different diseases, which would improve veterinary medicine.

In this study, 40 clinically relevant measurements were collected from 472 dogs of eight different breeds in five countries. The measurements included 9 morphometric measurements, 4 urinary and 24 clinical blood parameters, and 3 stress responses during the clinical exam (see Supplementary Table S1). We conducted breed specific GWAS and its meta-analysis with the 40 measurements in order to reveal the genetic contribution underlying individual differences.

## Results

### Breed-specific GWAS and meta-analysis

As shown in Supplementary Fig. S1, we showed a series of analyses conducted in this study. A total of 472 dogs were genotyped using a 170 K Illumina HD canine SNP array^19^ and 145,741 SNPs were selected after quality control (QC). We used the genome-wide efficient mixed model association (GEMMA) algorithm^20^ to perform association analyses of 301 combinations of phenotypes and breeds. We excluded 19 combinations due to missing phenotypes (Supplementary Table S1). The genomic inflation factor was 1.038 ± 0.006 (average ± standard error), suggesting that the algorithm was able to control for population structure. Supplementary Table S2 lists the seven genome-wide significant associations showing p < 5 × 10^−7^ determined by Bonferroni correction for the average number of common SNPs with minor allele frequency > 0.01 in each breed, although they did not show experimental wide significant association taking 301 tests into consideration. Supplementary Fig. S2 shows the association between the genotypes of a lead SNP and the clinical measurements.

In order to integrate the results from all eight breeds, meta-analyses were performed using the binary effects model to account for the existence of genetic effect in each breed^21^. This model assumes that (1) the effect may be present in some and absent in others of the studied breeds and (2) if the effect exists in a breed, the effect size is similar between breeds. The inflation factor was 1.140 ± 0.009. After applying a genomic control correction, 13 associations reached a genome-wide association level at p < 5 × 10^−7^ (Table 1). This number is much higher than expected under null hypothesis (2 = 40 × 0.05). The three associations for Alanine transaminase (ALT), fructosamine, and stress during physical examination reached an experiment-wide association level, accounting for multiple testing of the 40 phenotypes (p < 1.25 × 10^−8^).

**Table 1:** Significant associations in the trans-breed GWAS.

Phenotype	SNP	Chr	Position	P value	Experimental-wise p	M value								Human chr	Human position(Mb)	Nearest gene
						**BS**	**CK**	**DH**	**DM**	**FL**	**GS**	**LR**	**NF**			
Alanine transaminase	BICF2P496164	13	37,935,521	**1.02E-19**	**≪1E-10**	**1.000**	**1.000**	0.047	**1.000**	**0.959**	0.686	0.884	**1.000**	8	145.7	GPT, RECQL
Fructosamine	BICF2P1369335	17	61,926,412	**2.21E-11**	**8.85.E-05**	0.000	LowMAF	LowMAF	0.062	**0.949**	**1.000**	0.000	**0.999**	1	153.2	LELP1
Stress during physical exam	BICF2P1232291	1	109,530,837	**8.25E-09**	**0.032**	0.002	**1.000**	**0.997**	NoData	0.001	0.101	0.001	NoData	19	46.7	IGFL1
Morphometric parameter C (height at withers)	BICF2G630361702	3	91,114,590	5.47E-08	0.196	**1.000**	LowMAF	LowMAF	LowMAF	LowMAF	**1.000**	LowMAF	0.526	4	18.0	LCORL
Morphometric parameter C (height at withers)	BICF2S22921821	13	31,268,352	7.87E-08	0.270	0.003	0.097	**0.987**	0.861	**0.903**	**0.999**	**0.999**	0.376	8	136.3	KHDRBS3
C reactive protein	BICF2S2369445	31	15,294,994	1.76E-07	0.505	0.000	**0.991**	0.015	0.184	LowMAF	0.014	0.000	**1.000**	21	20.5	
Erythrocytes	BICF2P751898	32	14,312,867	2.49E-07	0.631	**0.982**	0.734	0.884	0.305	**1.000**	0.098	0.092	0.786	4	92.1	CCSER1
Morphometric parameter E (body length)	BICF2P67088	15	41,206,514	2.62E-07	0.649	0.003	LowMAF	**1.000**	0.501	0.753	LowMAF	**0.996**	0.201	12	102.8	IGF1
Glucose	BICF2S23023572	36	13,767,189	2.64E-07	0.652	**0.999**	0.653	0.811	0.700	**1.000**	0.870	**0.926**	0.819	2	169.8	ABCB11, G6PC2
Heart rate during clinical examination	BICF2P1178436	7	77,665,554	3.82E-07	0.783	0.219	**0.991**	**1.000**	0.249	0.287	LowMAF	0.257	NoData	4	110.6	CCDC109B
Stress during physical exam	BICF2G630755819	36	28,527,094	4.25E-07	0.817	**1.000**	0.670	0.808	NoData	0.561	**0.976**	0.829	NoData	2	187.1	
Morphometric parameter D (maximal chest diameter)	BICF2P200126	12	61,747,626	4.53E-07	0.837	0.001	LowMAF	**0.983**	0.534	0.004	0.720	**1.000**	0.499	6	104.7	
Stress during physical exam	BICF0218038	17	13,896,382	4.61E-07	0.842	0.001	**0.973**	**1.000**	NoData	0.658	0.039	0.090	NoData	2	18.8	NT5C1B

Associations with p < 5 × 10^−7^ is shown. The experimental-wise p values were calculated by the Bonferroni correction with the number of SNPs (n = 100,000) and phenotypes (n = 40). Adjusted P value for Alanine transaminase was not precisely calculated because its value is too smalll. The m-value was obtained using the binary effects model with Metasoft^21^. The chromosome position is based on CanFam 3.1; m-values > 0.9 suggesting the existence of genetic effect are underlined. BS, Belgian Shepherd; CK, Cavalier King Charles Spaniel; DH, Dachshund; DM, Doberman; FL, Finnish Lapphund; GS, German Shepherd; LR, Labrador Retriever; NF, Newfoundland. “NoData” and “LowMAF” indicate no phenotypic data and an MAF < 0.05 in that breed. The corresponding chromosome position in humans was defined according to hg19.

### Alanine transaminase: ALT

**Figure 1:**
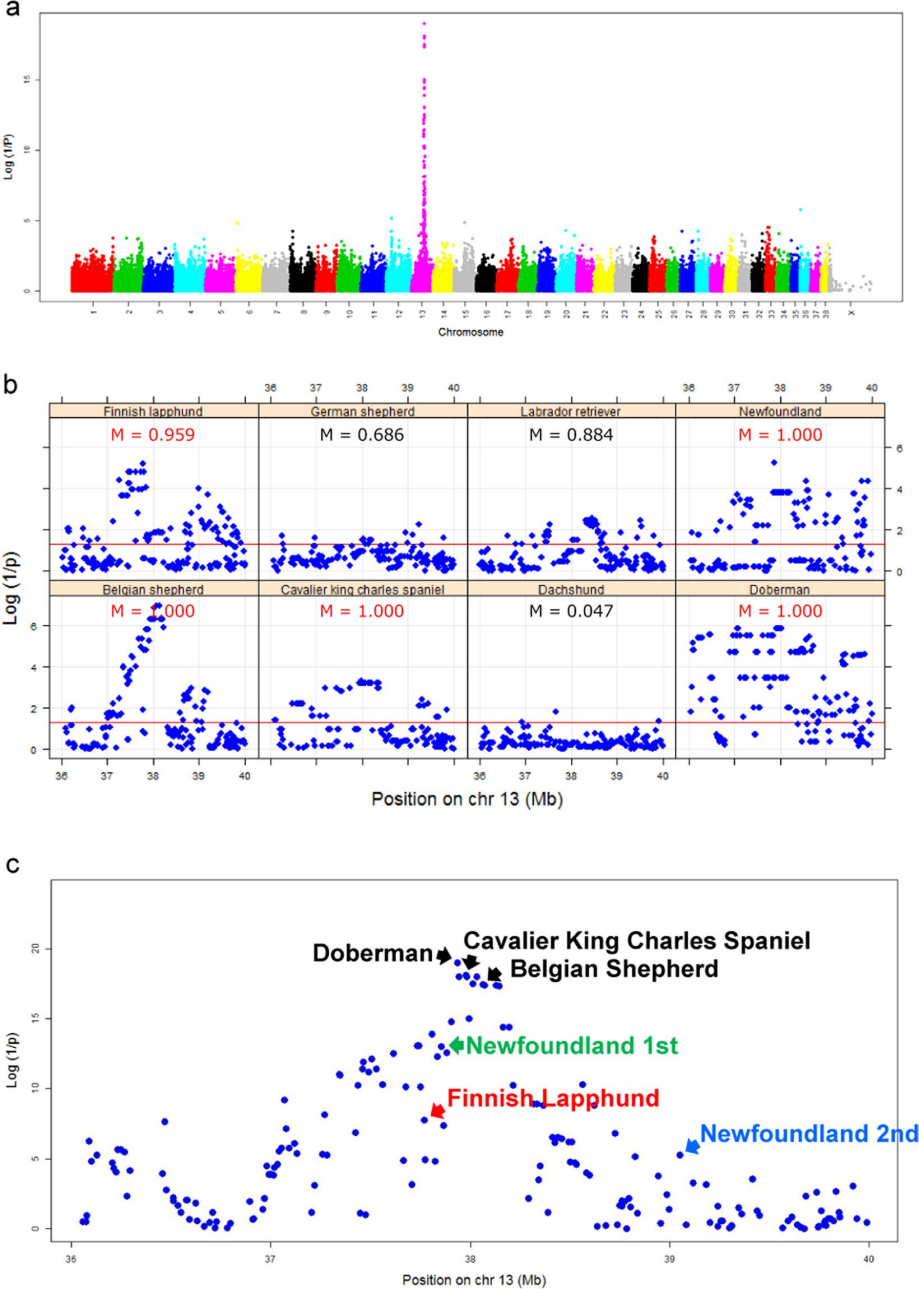
Results of a meta-analysis for ALT. (**a**) Manhattan plot of a meta-analysis (CanFam 3.1). **(b**) Local association profile for each breed covering 36–40 Mb of chromosome 13. The red line indicates p = 0.05. (**c**) Fig. 1c zoomed to the position 36–40 Mb of chromosome 13 in (**a**). Each dot shows the p value of the meta-analysis. We searched for the highest association of each breed separately. The three breeds with black font shared the same association signal, although lead SNPs were different and had high linkage disequilibrium (r^2^ > 0.973) with the lead SNP of the meta-analysis (BICF2P496164). Finnish Lapphunds with red color had an independent association signal, and Newfoundlands had two independent association signals colored in green and blue, because BICF2P496164 did not have high linkage disequilibrium with their lead SNPs in each breed.

ALT level in blood is used for the diagnosis of liver disease and injury in human and veterinary medicine^22^. The strongest signal (p = 1.02 × 10^−19^, experimental-wise p ≪ 1 × 10^−10^) for ALT was observed at BICF2P496164 (chr13: 37,935,521 on CanFam3.1) and we did not observe significant association in the other chromosomes. Manhattan plot and QQ plot are shown in Fig. 1a and Supplementary Fig. S3a. A plausible functional candidate gene, *GPT* encoding ALT, is located in this locus. A causal variant linked with the associated SNP would increase expression level of GPT, which would lead to the production of more ALT protein. Figure 1b shows the local association profile (p-values for all SNPs in the region) for each breed at this locus (the BICF2P496164 ± 2 Mb), which differed among the eight breeds. Indeed, the m-values calculated by meta-analysis using the binary effects model^21^ (Table 1) suggest a genetic effect of BICF2P496164 in five breeds only: Belgian Shepherds, Cavalier King Charles Spaniel, Doberman, Finnish Lapphund, and Newfoundland (m-value > 0.9). To identify additional independent associations in this locus, conditional analyses were performed with the lead SNP (BICF2P496164) as a covariate. BICF2P518128 (chr13: 36,960,246) showed independent association (P = 1.81 × 10^−5^) which passed threshold at P = 2.89 × 10^−4^ considering multiple testing with 173 SNPs in this locus.

**Figure 2:**
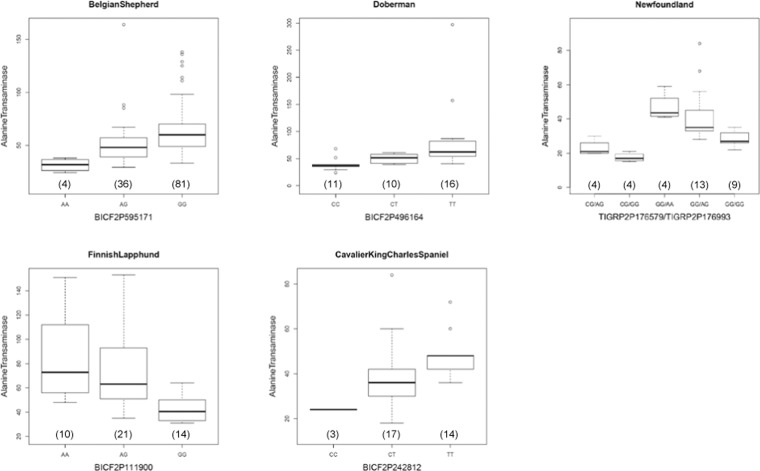
Association between the ALT values and genotypes of a lead SNP in each breed. The number of dogs for each genotype is shown in parenthesis.

In order to calculate the contribution of this locus for each breed, we searched for the highest association of each breed separately. As shown in Fig. 1c, Belgian Shepherd and Cavalier King Charles Spaniel had lead SNPs that differed from the lead SNP in the meta-analysis (BICF2P496164) but they had very high linkage disequilibrium with BICF2P496164 (r^2^ > 0.973). They were considered as one signal. In Finnish Lapphund, BICF2P111900 showed the highest association (P = 5.96 × 10^−6^) and moderate linkage disequilibrium with BICF2P496164 (r^2^ = 0.481). We considered BICF2P111900 as another signal. In Newfoundland, TIGRP2P176579 (P = 5.22 × 10^−6^) and TIGRP2P176993 (P = 1.91 × 10^−4^) showed independent associations and had low and moderate linkage disequilibrium respectively with BICF2P496164 (r^2^ = 0.075 and 0.544) as shown in Supplementary Fig. S4. They were considered two additional independent signals. In total, we observed four breed-specific signals. SNP(s) in this locus explained from 18.1 up to 47.7% of the variance (Table 2) based on the highest association of each breed. Association between each lead SNP and ALT is shown in the Fig. 2.

**Table 2:** Association between a lead SNP and the ALT level for each breed.

Breeds	SNP	Position (chr 13)	MAF	Pvalue	Contribution (%)
Belgian Shepherd	BICF2P595171	38,063,924	0.179	1.02.E-07	18.5
Doberman	BICF2P496164	37,935,521	0.421	1.43.E-06	47.7
Newfoundland	TIGRP2P176579	37,865,275	0.095	5.93.E-04	18.1
	TIGRP2P176993	39,050,399	0.369		
Finnish Lapphund	BICF2P111900	37,768,002	0.456	5.96.E-06	41.0
Cavalier King Charles Spaniel	BICF2P242812	37,978,745	0.338	5.00.E-04	31.2

There were two independent associations in Newfoundland.

### Fructosamine

**Figure 3:**
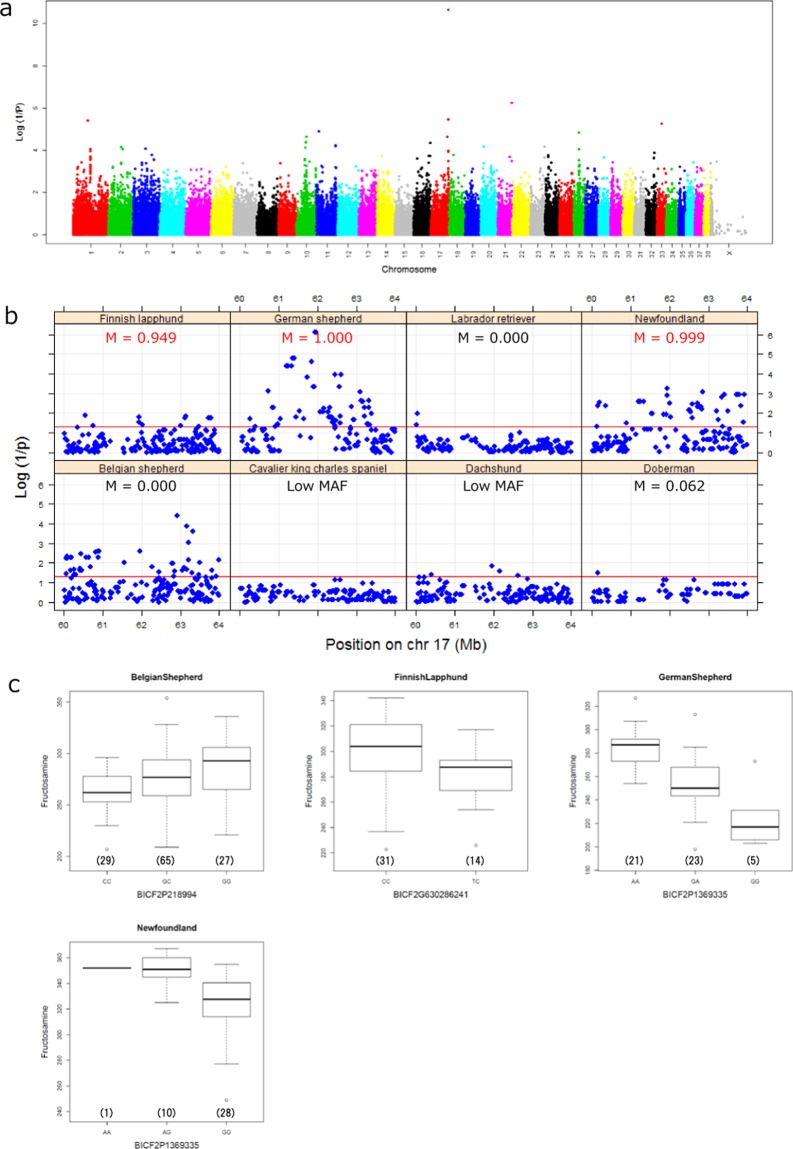
Results of a meta-analysis for fructosamine. (**a**) Manhattan plot of a meta-analysis (CanFam 3.1). (**b**) Local association profile for each breed covering 60–64 Mb of chromosome 17. The red line indicates p = 0.05. (**c**) Association between the frutosamine level and genotypes of a lead SNP in each breed. The number of dogs for each genotype is shown in parenthesis.

Fructosamine is a stable condensation product of glucose with serum proteins. It is used as a biomarker for diagnosing and managing diabetes because fructosamine reflects the average blood sugar concentration over the preceding 2 weeks^23^. The strongest signal (p = 2.21 × 10^−11^, experimental-wise p = 8.85 × 10^−5^) was observed at BICF2P1369335 (chr17: 61,926,412). LELP1 is the only protein coding gene located in this region but its association with fructosamine is not reported. Manhattan plot and QQ plot are shown in Fig. 3a and Supplementary Fig. S3b, respectively. Figure 3b shows the distribution of p-values for each breed at this locus (the lead SNP ± 2 Mb). The m-value suggests genetic effects of this SNP in Finnish Lapphund, German Shepherd and Newfoundland (Table 1). Although conditional analysis did not show independent association in these breeds, a p-value of 4.16 × 10^−5^ was detected in Belgian Shepherd dogs at a different SNP (BICF2P218994), which was 1 Mb from the lead SNP in the meta-analysis. Since linkage disequilibrium between BICF2P218994 and the lead SNP in the meta-analysis (BICF2P1369335) was low (r^2^ = 0.013), this is independent of the lead signal in the meta-analysis. This was also suggested by the M value of BICF2P1369335 in Belgium Shepherds (M = 0.000). Each lead SNP explained 11.2–45.8% of the variance as shown in Table 3. Figure 3c shows association between genotypes of each lead SNP and fructosamine.

**Table 3:** Association between a lead SNP and the fructosamine level for each breed.

Breeds	SNP	Position (chr 17)	MAF	Pvalue	Contribution (%)
Belgian Shepherd	BICF2P218994	62,923,275	0.4878	4.16.E-05	11.2
Finnish Lapphund	BICF2G630286241	60,538,765	0.1556	0.013	12.2
German Shepherd	BICF2P1369335	61,926,412	0.3367	7.34.E-07	45.8
Newfoundland	BICF2P1369335	61,926,412	0.1548	5.32.E-04	31.5

### Stress during physical examination

We identified experiment wide significant association of stress during physical examination (P = 8.25 × 10^−9^, experimental-wise p = 0.032) as shown in Table 1. The m-value suggests that the same QTL effect segregates in Cavalier King Charles Spaniel (P = 2.95 × 10^−6^, m-value = 1.000) and Dachshund (P = 1.51 × 10^−3^, m-value = 0.997) at BICF2P1232291 (chr 1: 109,530,837). Supplementary Table S3 show that in both breeds dogs with an A allele were considered to be more stressed during physical exam. There are no protein coding genes in this locus.

### Morphometric measurements

In the meta-analyses on morphometric measurements (Table 1 and Supplementary Fig. S5), four genome-wide associations were identified for the morphometric measurements C (height at withers), D (maximal chest diameter), and E (body length). Since they did not reach the experiment-wide significance level, a validation study with additional dogs is required. However, two regions, including *LCORL* on chromosome 3 and *IGF1* on chromosome 15, have previously been identified as being associated with breed differences in body size^24^. In the *LCORL* locus, the m-values calculated by the meta-analysis suggest that a genetic effect of this SNP exists only in Belgian Shepherd and German Shepherd. The SNP explained 26.8 and 35.4% of the variance, respectively (Supplementary Table S4). The association between Morphometric parameter C (height at withers) and the lead SNP (BICF2G630361702) in the *LCORL* locus in different breeds showed a clear pattern (Supplementary Fig. S6a). The largest and smallest breeds are fixed for opposite alleles.

The pattern between Morphometric parameter E (body length) and BICF2P67088 at the *IGF1* locus was less clear (Supplementary Fig. S6b) but the lead SNP was fixed to a C allele with a smaller value in the smallest breed, the Cavalier King Charles Spaniel. In the Dachshund, we observed another SNP (BICF2P274829) 440 kb away from BICF2P67088 showing the highest association in this locus (p < 1.44 × 10^−5^). This SNP explained 68.1% of the variance in morphometric parameter E (body length) in Dachshunds. The length of the back in dogs with the GG genotype was 12.6% (5.9 cm) longer than that in dogs with the AA genotype (GG: 52.3 cm; AA: 46.4 cm, Supplementary Fig. S7).

Supplementary Table S4 and Supplementary Fig. S7 showed other associations between phenotypic values and genotypes of a lead SNP in significant loci identified in the meta-analyses.

## Discussion

In this study, we collected 40 clinically relevant measurements from 472 healthy dogs of eight breeds, and performed breed-specific GWAS and meta-analyses. In total, 13 genome-wide significant associations were identified in meta-analyses. This number was much higher than expected under the null hypothesis. We observed experiment-wise significant association for ALT, fructosamine, and stress during physical exam.

Of these, the strongest association was for ALT. This association has been reported in a GWAS with 353 dogs consisting of multiple breeds^25^. Taking advantage of the possibility to analyze each dog breed in our study, we investigated a lead SNP in each breed. We have identified four different signals (Fig. 1c). This locus in each dog breed contributed from 18.1% up to 47.7% of the variance of the enzyme range (Table 2). Since these lead SNPs most probably do not represent causative variants, as illustrated in human GWASs^26^, the contribution from this locus could even be greater. In humans, the heritability of ALT was estimated at 0.48^27^ and a GWAS of ALT using 61,089 individuals have identified four loci^28^ but not the locus which corresponds to the canine locus identified here. In the human study, the effect size rather than the contribution was used as an indicator of the genetic effects of a single variant. The effect size for the four loci was 0.016‒0.060 in humans whereas it reached 1.08‒1.37 in the five dog breeds from this study. Although effect size calculated from GWAS are known to be affected by a bias as “winner’s curse”, the change in the concentration of ALT per copy of each effect allele is roughly more than ten-fold higher in dogs than in humans. ALT is a liver specific cytosolic enzyme that is routinely screened for health assessment as a sensitive indicator for hepatocellular injury in dogs. Significant ALT variation exists among dog breeds^29^ and it would be important to know the contribution of a lead SNP in each breed to improve interpretation of ALT value in veterinary medicine especially in breeds predisposed to liver disease.

Individual differences in the level of fructosamine were mapped to chromosome 17. As in ALT, association was not found in all breeds but in only three of the studied breeds. One SNP in this locus also explained a large proportion (11.2–45.8%) of the variance. Note that the previous GWAS focusing on fructosamine data of the same dogs did not identify any genome wide significant associations^29,30^. This difference could be explained by different analyses methods. The previous study used linear mixed models including a fixed effect for breed, while here we normalized raw phenotype values to minimize the effect of outliers for statistical analysis, used the GEMMA algorithm in each breed separately, and merged p values by binary effects model to account for existence of genetic effect (see Materials and Methods). Note that using the same data, a genome wide significant association on chromosome 3 (P = 1.68 × 10^−6^, P for permutation = 0.024) was reported in Belgium Shepherd^30^. In the current analysis, we observed a similar tendency, but it was not significant (P = 5.15 × 10^−6^). This divergence could be explained by different analysis methods and slight difference in the number of dogs (n = 118 in the previous analysis and n = 113 in this study).

We identified an experimental-wide association with stress during physical examination at chromosome 1. Two breeds, Cavalier King Charles Spaniels and Dachshunds, showed the same tendency. Dogs with the A allele of BICF2P1232291 showed more stress during physical examination. The research field of the genetics of dog behavior trait is of much interest, because dogs are unique animal models that can be used to understand behavioral traits^31^. However, there are a limited number of studies^32,33^, because assessment of behavioral traits is a difficult task. In this study, we used subjective judgment from dog handlers. Although experienced dog handlers might provide better assessment than other behavioral tests, handler assessment should be replaced by a method to assess endophenotype, which has the potential to measure the same phenotype anywhere and to dissect behavior into biological response^31^.

A large contribution due to a single variant was also observed for other phenotypes. Since these associations should be validated by using independent samples, we only focus on associations that can be supported by additional evidence. Morphometric parameters C (height at withers) and E (body length) were mapped to regions close to *LCORL* and *IGF1*, respectively. These regions have already been reported to be associated with breed differences in body size^19,24,34^. The lead SNP reported here contributed to individual differences in morphometric parameters in a few breeds only because it was fixed for the other allele in some breeds (Supplementary Fig. S6). Again the contribution of each SNP was larger than observed in human. Any one SNP associated with these morphologic traits could explain 6.8–68.1% of the variance within a breed (Supplementary Table S4). In comparison, all 697 variants identified in the latest GWAS explain only ~16% of the phenotypic variance in human height^35^.

A large contribution of a single variant was also observed for the glucose level. Glucose serum level in this study was linked to a locus of chromosome 36 that corresponds to the locus associated with fasting plasma glucose level in humans^36^, close to the gene *G6PC2*. *G6PC2* encodes an enzyme that catalyzes the hydrolysis of glucose-6-phosphate, allowing the release of glucose into the bloodstream. While homozygotes of either allele showed differences of only 2.7% in glucose level (5.24 and 5.10 mmol/l) in humans, they showed differences of 26.6% (1.067 and 0.843 mmol/l) in Finnish Lapphund dogs. Further study is needed to reveal if this SNP influences susceptibility for diabetes.

This study has three main implications for canine GWAS. (1) International collaboration for sampling allows increasing the number of dogs to get a higher statistical power. However, although we have collected phenotypes from the same breed with the same sex in neighboring countries we have observed clear differences in the phenotypes (Supplementary Fig. S8 for female Labrador Retriever and S9 for male Belgian Shepherd). Environmental effects, diagnostic procedure, breeding selection for specific purpose^37^, or other unrecognized phenotyping biases, can cause this kind of “group effect”^38^. So we had to normalize data within each group instead of merging the original phenotyping data from different groups to avoid this bias of “group effect”. (2) GWAS using dogs of different breeds^25,39,40^ can efficiently map the chromosome region associated with a phenotype. However, the association identified by this kind of analysis is not always applicable to all breeds. Indeed, an association between height and the *LCORL* locus was found by GWAS using dogs of different breeds^24^ but in our study the lead SNP contributed to individual difference only in 2 of 8 breeds and the allele was not polymorphic in four breeds. The same is true for ALT, fructosamine and other phenotypes. Therefore, in the context of a genetic marker for the improvement of veterinary medicine, the identification of SNPs for individual differences rather than breed differences is important. It is necessary to analyze the association in each dog breed using a sufficient number of dogs. (3) In this study, we identified three experiment-wide significant associations whose SNPs explained a much larger part of the variance than that in humans. However, the current number of dogs might not have had enough power to identify other associations with smaller effect sizes. Supplementary Table S5 shows the results of a power calculation to have genome wide significance at P = 5 × 10^−7^ under different scenarios of effect size (0.01–1.5) and total sample size (50 – 10,000) with GPower 3.1^41^. The total number of dogs in this study (n = 472) was enough for sufficient statistical power to identify the strongest signal for ALT, because its effect size was 1.08–1.37. However, when effect size is 0.5, for example, we need more than 1,000 dogs, and the current number of dogs is not enough for sufficient statistical power. Therefore, we need more dogs, or we need to perform a meta-analysis with other studies examining the same phenotypes in order to identify weaker associations.

In summary, the meta-analyses enabled the identification of 13 genome-wide associations for clinical measurements. Most of the associated phenotypes were strongly controlled by only one or two genetic variants at a single locus. The genetic effect of a single variant was much larger than in humans. This study illustrates the importance of performing meta-analysis after breed-specific GWAS to reveal genetic contribution to individual differences in clinical measurements, which would lead to improvement of veterinary medicine.

## Methods

### Dogs

A total of 472 healthy dogs consisting of eight breeds (Belgian Shepherd, Cavalier King Charles Spaniel, Dachshund, Doberman, Finnish Lapphund, German Shepherd, Labrador Retriever, and Newfoundland) from five countries (Belgium, Denmark, Finland, France, and Sweden) were recruited for this study as part of the EU-funded LUPA project (Supplementary Table S6)^42^. Our group previously reported GWAS focusing only on fructosamine using the same dogs^30^. However, using the binary effects model that was expected to increase the statistical power of the analysis, we have obtained a new association regarding fructosamine that is worth being reported in the present study.

To be included in the study, dogs had to be pure-bred, healthy and between 2 and 7 years of age with a normal body condition score. Dogs could not be related to each other at the parental level. Within each group and each breed, dogs had to be of the same sex; females were in anoestrus or spayed, males were intact. Exclusion criteria consisted of any finding indicating systemic or organ-related disease in the history, physical examination, blood work or urinalysis described below.

### Clinical data collection

We collected 40 clinical relevant measurements as shown in Supplementary Table S1. All dogs were examined fasted (12 hours for food and 2 hours for water). A complete history was taken and each dog underwent a thorough physical examination. During this physical exam several morphometric parameters and stress responses were measured as well. Stress phenotype was assessed by the same dog handler within each country (5 dog handlers in total) using the same 4-criteria scale (stress absent, mild stress, moderate stress and heavy stress). This scale was subjective, and though handler differences were not controlled, all of them were experienced vets that were used to interact with dogs. Furthermore, within breeds, the same handler performed the tests on all dogs, except for Labradors and Belgian Shepherds, which were shared between different dog handlers.

Blood were taken to perform routine analysis, as well as to measure cardiovascular hormones and biochemical parameters related to metabolic syndrome. Urine sample was collected by voiding on each dog. Blood sampling was carried out by venipuncture and blood was collected into EDTA and serum tubes. Routine analysis of hematology and biochemistry including total protein, parameters of liver and kidney function and serum electrolyte concentrations were performed. Standard urine analysis was performed by dipstick chemistry test and refractometer for urine specific gravity. Serum was collected to measure parameters related to lipid (free fatty acid, cholesterol, triglycerides and C reactive protein) and carbohydrate metabolism (glucose, fructosamine and insulin), as well as cortisol level. Plasma was collected to measure cardiovascular hormones (endothelin-1, aldosterone, plasma renin activity, NT-proANP and NT-proBNP). Tubes were centrifuged within 30 minutes of blood sample collection. Plasma and serum were harvested, transferred into plastic cryotubes and samples were frozen and stored at −80 °C. All samples were later transported frozen to different accredited laboratories. All analyses were performed using commercially available assays validated for dogs, according to manufacturers’ instructions. Insulin, cortisol and cardiovascular hormones samples were analyzed in duplicate by personnel blinded to dog identity, and the mean of the two results was used for data analysis. Data were tested for normality using Shapiro Wilk test. Outliers were individually checked and some of them were excluded from the analysis if their results seemed inappropriate.

Descriptive statistics for the 40 phenotypes are listed in Supplementary Table S1. As breed differences for some phenotypes have been reported elsewhere^43,44^, breed and sex differences for these phenotypes were expected. But we have also observed differences between countries where Labrador retriever and Belgian Shepherd were sampled. Among the 35 phenotypes collected in female Labrador Retrievers in Denmark and France significant differences were observed in 10 phenotypes (p < 1.43 × 10^−3^ = 0.05/35) as shown in the Supplementary Fig. S8. Similarly 18 of 36 phenotypes showed significant differences (p < 1.39 × 10^−3^ = 0.05/36) between Belgium and France for male Belgian Shepherds (Supplementary Fig. S9). Therefore, we decided to normalize phenotype data within each group based on country, breed, and sex before GWAS to eliminate these kinds of group effects.

We converted a raw value to a relative rank within each dog group for the following investigations for the purpose of conducting nonparametric analyses of the phenotypic data. To calculate the relative rank of each phenotype within each dog group, each dog was assigned a rank, which was divided by the number of dogs in that group. The relative rank was thus between 0 and 1.

### Genotyping

Genotypic analyses were conducted using 472 dogs with a 170 K Illumina canine HD SNP array^19^. The CanFam 3.1 reference sequence was used. The QC process eliminated 38,725 SNPs due to a low call rate (<95%), low minor allele frequency (MAF) (<0.01), and deviation from Hardy-Weinberg equilibrium (p < 1 × 10^−6^ for each breed). Finally, 145,741 SNPs were analyzed in this study.

A total of 56,811 SNPs with an MAF > 0.2 and mutual r^2^ < 0.5 according to the indep-pairwise function in PLINK 1.07^45^ (–indep-pairwise 1000 100 0.5) were selected for principal component analysis (PCA). The analysis was performed using Eigenstrat 5.0.1^46^ to check outliers inconsistent with the recorded dog breeds. A PCA did not reveal any outliers that were inconsistent with the recoded dog breeds (Supplementary Fig. S10).

### Breed-specific GWAS and meta-analysis

Before GWAS, we adjusted confounding effects of breed and sex by normalizing phenotypes separately within each breed and sex. In GWAS, we did not include any covariates. The GEMMA algorithm was applied to perform association analyses for a single phenotype in each breed accounting for population stratification^20^. The genomic inflation factor (λ) was defined as the ratio of the median of the observed test statistic to the expected median. When λ > 1, population stratification was suggested. We divided the test statistic of each SNP by λ as a corrected test statistic and recalculated a corrected P value as a genomic control correction. We set threshold for genome wide significance at p = 5 × 10^−7^ because there were 103,740 common SNPs in each breed on average. The contribution of a single variant to explain the variance of each relative rank was calculated^47^. This method provides a bias-corrected estimate of the proportion of phenotypic variance.

A meta-analysis was also performed to integrate the p-values from the eight breeds for each phenotype. This analysis is similar to a trans-ethnic GWAS of humans that was used to examine several complex diseases such as type 2 diabetes^48^. A new framework, the binary effects model, was developed to account for the existence of effect to improve the statistical power as compared with fixed effects and random effects models^21^. This model also provides the posterior probability that the effect could exist in each breed as an m-value. An m-value > 0.9 suggests existence of a genetic effect in that breed.

We used the Metasoft ver. 2.01 to combine the association results in each breed with the binary model. The binary effects model was applied to the 40 phenotypes with SNPs with MAF ≥ 0.05 in two or more breeds. When the genomic inflation factor was >1, a genomic control correction was applied. The same threshold at p = 5 × 10^−7^ was used for genome-wide significance, and p = 1.25 × 10^−8^ (= 5 × 10^−7^/40) was defined as the threshold value for experiment-wide significance. Since the lead SNP in a meta-analysis does not always show the highest association in a breed-specific GWAS, the best association was selected within ±2 Mb of a lead SNP for each dog breed. The regional association threshold was set by Bonferroni correction using the number of SNPs in that locus. We investigated independence between the lead SNP in the meta-analysis and the lead SNP in each breed by the calculation of r^2^ in each breed separately.

### Ethics statement

This was already described in the previous paper^30^. The study was carried out in accordance with the recommendations in the Guide for the Care and Use of Laboratory Animals of the National Institutes of Health and approved by an ethical Committee in Belgium (Commission d’Ethique Animale, Université de Liegè, Belgium, permit number 754), Sweden (Uppsala Local Ethical Committee, Uppsala, Sweden, permit number C 115/8), Finland (Viikki Campus Research Ethics Committee, Helsinki, Finland, no approval number used by committee) and Denmark (Local Ethical Committee, University Hospital for Companion Animals, Copenhagen, Denmark, at the time of the approval, the committee did not operate with approval numbers). In France, data was obtained before the creation of the Local Ethical Committee dedicated to clinical research (ComERC-ENVA). As the data were from client owned dogs undergoing normal veterinary exams, there was no animal experiment according to legal definitions in France. However, all local regulations related to clinical procedures were observed. In all countries, informed owners consent was obtained for use of samples and data for scientific research. All undertaken procedures were part of routine veterinary clinical exam in all countries and the local responsible co-investigator, who was also licensed to practice veterinary medicine within the EU, was responsible for collection of the samples.

## Supplementary information

Supplementary information.

## Data Availability

All genotyping data was deposited in DRYAD (https://datadryad.org/stash/dataset/doi:10.5061/dryad.ft6fv).
