# Supplementary material for: Genome wide association study of 40 clinical measurements in eight dog breeds
Source: Sci Rep. 2020 Apr 16;10:6520. doi: 10.1038/s41598-020-63457-y (PMC7162946; doi:10.1038/s41598-020-63457-y)

Supplementary Information

Genome wide association study of 40 clinical measurements in eight dog breeds

Yukihide Momozawa[1,2,#,*], Anne-Christine Merveille[1,3,#], Géraldine Battaille[1,3], Maria Wiberg[4], Jørgen Koch[5], Jakob Lundgren Willesen[5], Helle Friis Proschowsky[6], Vassiliki Gouni[7], Valérie Chetboul[7], Laurent Tiret[8], Merete Fredholm[5], Eija Seppälä[9], Hannes Lohi[9], Michel Georges[1,3], Anne-Sophie Lequarré[1,3,*]

[#]Equally contributed

[1]Unit of Animal Genomics, GIGA Institute, University of Liège, Liège, Belgium.

[2]Laboratory for Genotyping Development, RIKEN Center for Integrative Medical Sciences, Kanagawa 230-0045, Japan

[3]Department of Clinical Sciences, Faculty of Veterinary Medicine, University of Liège, Belgium.

[4]Department of Equine and Small Animal Medicine, Faculty of Veterinary Medicine, University of Helsinki, Finland

[5]Faculty of Health and Medical Sciences, University of Copenhagen, 1870 Frederiksberg C, Denmark

[6]The Danish Kennel Club, 2680 Solrød Strand, Denmark

[7]Cardiology Unit (UCA), Alfort Veterinary University Hospital, National Veterinary School of Alfort (ENVA), France

[8] U955 - IMRB, Biology of the neuromuscular system, Inserm, National Veterinary School of Alfort (ENVA), France

[9] Department of Veterinary Biosciences, Department of Medical and Clinical Genetics, University of Helsinki, Folkhälsan Research Center, Helsinki, Finland

Supplementary Table S1: Average and standard error of all 40 phenotypes for each group of dogs by breed and country

Phenotype			Breed	Belgian shepherd		Cavalier king charles spaniel	Dachshund	Doberman	Finnish lapphund	German shepherd	Labrador retriever			Newfoundland
			Country	Belgium	France	Sweeden	Finland	France	Finland	Finland	Denmark	France	Sweeden	Denmark
Abbreviation	Phenotype	Unit	Sex	Male	Male	Male	Male	Male	Male	Male	Female	Female	Male	Female
[Morphometric measurements]														
BC	Body score (1-9)			4.1 ± 0.1	5.0 ± 0.0	5.4 ± 0.1	3.8 ± 0.2	5.0 ± 0.1	3.8 ± 0.2	3.6 ± 0.1	5.6 ± 0.1	5.4 ± 0.1	5.5 ± 0.1	5.6 ± 0.1
WG	Weight	kg		31.5 ± 0.5	29.4 ± 0.8	9.4 ± 0.3	10.2 ± 0.4	42.9 ± 0.5	20.0 ± 0.5	36.1 ± 0.5	28.6 ± 0.5	30.2 ± 0.6	31.0 ± 0.7	54.0 ± 0.9
MOR_A	Morphometric parameter A (Snout length)	cm		11.2 ± 0.1	11.4 ± 0.3	3.9 ± 0.1	8.6 ± 0.1	12.5 ± 0.2	8.1 ± 0.1	11.2 ± 0.1	8.8 ± 0.1	9.4 ± 0.2	9.2 ± 0.1	9.6 ± 0.1
MOR_B	Morphometric parameter B (Head length)	cm		17.4 ± 0.1	14.1 ± 0.4	9.2 ± 0.2	13.1 ± 0.2	17.2 ± 0.2	14.2 ± 0.1	16.8 ± 0.2	12.6 ± 0.1	15.0 ± 0.2	13.8 ± 0.2	14.4 ± 0.2
MOR_C	Morphometric parameter C (Height at withers)	cm		68.3 ± 0.4	62.2 ± 0.7	35.2 ± 0.3	26.4 ± 0.4	71.6 ± 0.3	50.2 ± 0.4	64.8 ± 0.4	54.8 ± 0.3	54.9 ± 0.4	59.0 ± 0.5	67.5 ± 0.5
MOR_D	Morphometric parameter D (Maximal chest diameter)	cm		74.3 ± 0.4	54.1 ± 1.3	50.2 ± 0.5	47.7 ± 0.6	69.5 ± 1.1	62.2 ± 0.6	76.0 ± 0.5	54.7 ± 0.5	55.9 ± 1.3	73.2 ± 0.7	67.3 ± 0.7
MOR_E	Morphometric parameter E (Body length)	cm		69.5 ± 0.5	69.2 ± 1.0	43.8 ± 0.5	48.9 ± 0.6	71.7 ± 0.3	56.4 ± 0.7	71.3 ± 0.7	61.0 ± 0.6	62.2 ± 0.9	65.2 ± 0.6	79.1 ± 0.7
MOR_F	Morphometric parameter F (Height at point of shoulder)	cm		45.9 ± 0.3	45.9 ± 1.1	19.7 ± 0.3	16.0 ± 0.6	50.4 ± 0.6	34.6 ± 0.4	45.8 ± 0.4	40.1 ± 0.5	37.9 ± 0.9	36.9 ± 0.5	50.3 ± 0.8
MOR_G	Morphometric parameter G (Height at olecranon)	cm		35.8 ± 0.3	35.9 ± 0.7	16.4 ± 0.3	9.1 ± 0.4	37.6 ± 0.6	29.5 ± 0.4	36.4 ± 0.3	27.5 ± 0.4	29.0 ± 0.7	30.8 ± 0.4	32.1 ± 0.4
[Urinary measurements]														
UR_SG	Urine specific gravity			1043.3 ± 1.4	1041.1 ± 2.7	1042.7 ± 1.7	1046.8 ± 1.2	1040.1 ± 2.2	1038.9 ± 1.6	1043.3 ± 1.2	1028.0 ± 2.1	1034.2 ± 3.2	1035.9 ± 1.7	1029.4 ± 1.7
UR_PROT	Amount of protein in urine sample			1.0 ± 0.1	0.1 ± 0.1	1.7 ± 0.1	1.8 ± 0.1	0.8 ± 0.2	1.7 ± 0.1	1.4 ± 0.1	1.3 ± 0.2	0.1 ± 0.1	1.3 ± 0.1	1.3 ± 0.2
UR_PH	pH of urine sample			6.2 ± 0.1	5.9 ± 0.2	6.1 ± 0.2	6.6 ± 0.1	6.2 ± 0.2	7.0 ± 0.1	6.9 ± 0.1	7.6 ± 0.1	6.5 ± 0.2	5.8 ± 0.1	7.5 ± 0.1
UR_BIL	Amount of bilirubin in urine sample			1.0 ± 0.1	0.1 ± 0.1	0.2 ± 0.1	1.5 ± 0.2	0.1 ± 0.1	1.2 ± 0.1	1.5 ± 0.1	2.8 ± 0.3	0.0 ± 0.0	0.7 ± 0.1	NA
[Clinical blood measurements]														
BA_HB	Hemoglobin	g/dL		17.8 ± 0.2	16.2 ± 0.3	13.7 ± 0.2	16.4 ± 0.3	17.2 ± 0.3	17.1 ± 0.2	16.8 ± 0.2	15.2 ± 0.3	15.0 ± 0.3	15.6 ± 0.2	15.1 ± 0.2
BA_ERY	Erythrocytes	10^6/ml		7.5 ± 0.1	7.0 ± 0.2	5.7 ± 0.1	6.9 ± 0.1	7.5 ± 0.1	7.2 ± 0.1	7.3 ± 0.1	6.6 ± 0.1	6.4 ± 0.2	6.5 ± 0.1	6.3 ± 0.1
BA_HCT	Hematocrite	%		51.9 ± 0.5	48.1 ± 0.9	40.4 ± 0.6	48.2 ± 1.0	51.1 ± 0.8	51.3 ± 0.6	50.1 ± 0.4	45.2 ± 0.8	44.3 ± 1.2	46.0 ± 0.5	43.3 ± 0.6
BA_LEUCO	Leucocytes	/ml		8688.6 ± 221.2	10020.0 ± 426.0	10997.1 ± 394.2	8943.8 ± 465.8	10388.4 ± 316.8	8453.6 ± 337.6	9561.2 ± 304.5	8927.3 ± 347.7	9470.7 ± 465.0	8104.8 ± 299.5	8798.5 ± 416.4
BA_BUN	Blood urea nitrogen	mmol/l		12.1 ± 0.4	6.0 ± 0.2	4.6 ± 0.2	5.5 ± 0.4	5.4 ± 0.2	5.6 ± 0.2	6.5 ± 0.2	4.6 ± 0.2	5.0 ± 0.3	5.3 ± 0.2	4.6 ± 0.2
BA_CREAT	Creatinin	µmol/l		79.4 ± 1.3	89.9 ± 3.1	56.5 ± 1.5	72.6 ± 1.2	76.7 ± 1.7	94.1 ± 1.5	98.0 ± 1.4	95.9 ± 2.2	92.8 ± 2.5	87.3 ± 1.7	90.3 ± 2.1
BA_AP	Alkaline phosphatase	UI/l		43.3 ± 2.3	156.5 ± 10.7	50.2 ± 4.1	56.8 ± 3.7	177.1 ± 24.9	78.0 ± 3.1	55.8 ± 3.1	105.1 ± 8.2	95.6 ± 7.7	83.4 ± 3.9	132.2 ± 9.4
BA_AT	Alanine transaminase	UI/l		61.6 ± 2.4	52.5 ± 3.8	40.6 ± 2.4	85.1 ± 9.2	61.7 ± 7.6	65.2 ± 4.6	54.3 ± 2.5	55.5 ± 3.2	54.5 ± 5.2	55.8 ± 2.7	34.3 ± 2.6
BA_TP	Total protein	g/l		70.3 ± 1.0	62.1 ± 1.0	56.4 ± 0.7	58.1 ± 0.7	64.7 ± 0.8	61.5 ± 0.5	59.7 ± 0.4	59.2 ± 0.7	59.1 ± 0.9	59.0 ± 0.7	64.0 ± 0.6
BA_NA	Sodium	mEq/l		148.4 ± 0.3	144.4 ± 0.6	148.4 ± 0.6	148.4 ± 0.3	143.3 ± 0.5	150.2 ± 0.3	148.7 ± 0.2	148.8 ± 0.3	143.9 ± 0.5	150.5 ± 0.6	149.5 ± 0.5
BA_K	Potassium	mEq/l		4.8 ± 0.0	4.0 ± 0.1	4.4 ± 0.1	4.2 ± 0.0	4.0 ± 0.0	4.3 ± 0.0	4.4 ± 0.0	4.4 ± 0.1	4.1 ± 0.1	4.5 ± 0.0	4.5 ± 0.1
PRA	Plasma renin activity	ng/ml/h		1.0 ± 0.2	0.9 ± 0.1	0.8 ± 0.1	1.5 ± 0.1	0.7 ± 0.1	0.6 ± 0.1	0.8 ± 0.1	1.0 ± 0.1	0.6 ± 0.1	0.9 ± 0.1	0.7 ± 0.1
ALDO	Aldosterone	pg/ml		23.3 ± 3.9	32.0 ± 7.2	37.4 ± 6.1	72.6 ± 15.3	40.3 ± 8.4	51.1 ± 5.4	40.7 ± 7.8	68.2 ± 6.9	27.3 ± 5.4	71.5 ± 10.7	49.5 ± 5.1
ENDO_COR	Corrected endothelin	pg/ml		1.2 ± 0.1	1.3 ± 0.1	1.0 ± 0.1	1.2 ± 0.1	0.9 ± 0.1	1.7 ± 0.1	1.4 ± 0.1	1.8 ± 0.1	1.3 ± 0.1	1.5 ± 0.1	3.5 ± 0.2
NT-proANP	N-terminal pro-atrial natriuretic peptide	fmol/ml		929.2 ± 29.7	NA	1032.2 ± 50.2	NA	NA	NA	NA	NA	NA	998.5 ± 27.9	NA
NT-proBNP	N-terminal pro-brain natriuretic peptide	pmol/l		594.8 ± 47.0	NA	603.8 ± 43.6	NA	NA	NA	NA	NA	NA	716.3 ± 64.8	NA
CHOLEST	Cholesterol	g/l		2.0 ± 0.1	2.4 ± 0.1	1.9 ± 0.1	1.5 ± 0.1	2.0 ± 0.1	2.2 ± 0.1	2.0 ± 0.1	2.1 ± 0.1	1.9 ± 0.1	2.1 ± 0.1	2.6 ± 0.1
TG	Triglycerides	g/l		0.4 ± 0.0	0.5 ± 0.1	0.5 ± 0.1	0.4 ± 0.0	0.6 ± 0.1	0.5 ± 0.0	0.4 ± 0.0	0.5 ± 0.0	0.5 ± 0.0	0.4 ± 0.0	0.6 ± 0.0
GLUC	Glucose	g/l		1.1 ± 0.0	1.0 ± 0.0	0.9 ± 0.0	1.0 ± 0.0	1.0 ± 0.0	1.0 ± 0.0	0.9 ± 0.0	1.0 ± 0.0	1.0 ± 0.0	0.9 ± 0.0	1.0 ± 0.0
FRUCTO	Fructosamine	µmol/l		279.9 ± 2.7	260.6 ± 5.4	278.4 ± 5.3	257.1 ± 5.5	260.4 ± 7.0	295.3 ± 4.2	264.2 ± 4.0	313.2 ± 4.1	277.1 ± 6.5	320.7 ± 3.3	330.8 ± 4.0
FFA	Free fatty acid	mmol/l		0.8 ± 0.0	0.9 ± 0.1	0.8 ± 0.1	0.9 ± 0.1	1.0 ± 0.1	0.7 ± 0.1	0.7 ± 0.1	1.0 ± 0.1	0.9 ± 0.1	1.0 ± 0.0	0.9 ± 0.1
CRP	C reactive protein	mg/l		2.0 ± 0.1	2.7 ± 0.3	2.0 ± 0.2	2.4 ± 0.3	2.2 ± 0.2	2.0 ± 0.1	2.3 ± 0.1	3.0 ± 0.2	2.5 ± 0.3	2.1 ± 0.2	3.1 ± 0.2
INS	Insulin	mU/l		21.0 ± 0.7	15.6 ± 0.9	18.2 ± 1.5	17.5 ± 1.5	23.0 ± 1.1	25.0 ± 1.5	16.2 ± 0.8	14.7 ± 0.7	16.7 ± 0.7	16.8 ± 0.7	19.6 ± 1.1
CORTISOL	Cortisol	nmol/l		61.7 ± 5.6	34.8 ± 4.9	44.3 ± 3.5	43.4 ± 4.9	50.9 ± 5.5	96.6 ± 8.7	70.4 ± 7.5	102.7 ± 10.6	40.6 ± 7.1	59.5 ± 6.2	68.8 ± 6.4
[Stress responses during these measurements]														
HR	Heart rate during clinical exam	/min		106.8 ± 1.8	96.8 ± 4.1	116.1 ± 4.1	95.8 ± 3.0	105.5 ± 3.9	86.8 ± 2.5	73.1 ± 2.0	NA	95.2 ± 4.7	85.3 ± 3.3	NA
STR_PE	Stress during physical exam (1-4)			2.3 ± 0.1	NA	1.4 ± 0.1	2.1 ± 0.1	NA	1.9 ± 0.1	2.2 ± 0.1	NA	NA	1.7 ± 0.1	NA
BD_STR	Stress during blood sampling (1-4)			2.1 ± 0.1	NA	1.9 ± 0.1	NA	NA	NA	NA	NA	NA	1.8 ± 0.1	NA

Supplementary Table S2: Significant associations in the breed-specific GWAS

Breed	Phenotype	SNP	Chr	Position	MAF	P value	Experimental -wise p	Contribution(%)	Human chr	Human position (Mb)	Nearest gene
Belgian Shepherd	Morphometric parameter C (height at withers)	BICF2G630361674	3	91,085,475	0.342	3.74E-08	0.676	26.8	4	18.1	LCORL
Belgian Shepherd	Morphometric parameter F (height at point of shoulder)	BICF2G630361674	3	91,085,475	0.342	7.17E-08	0.885	22.7	4	18.1	LCORL
Belgian Shepherd	Alanine transaminase	BICF2P595171	13	38,063,924	0.179	1.02E-07	0.954	18.5	8	146.0	GPT
Labrador Retriever	Triglycerides	BICF2G63099748	25	31,202,347	0.470	1.52E-07	0.990	23.3	8	26.2	PPP2R2A
Cavalier King Charles Spaniel	NT-proANP	TIGRP2P312306	24	17,594,307	0.214	1.52E-07	0.990	62.6	20	3.7	C20orf27
Labrador Retriever	Blood urea nitrogen	BICF2P419856	13	43,594,553	0.168	2.67E-07	1.000	24.0	4	47.7	CORIN
Labrador Retriever	Plasma renin activity	BICF2G630689266	18	54,483,554	0.250	2.98E-07	1.000	18.5	11	61.7	BEST1

The chromosome position is based on CanFam 3.1. The p-value was corrected using the genomic control. The experimental-wise p values were calculated by the Bonferroni correction with the number of SNPs

Supplementary Table S3: Association between genotypes of BICF2P1232291and stress during physical exam

	Stress during physical exam					
	Cavalier King Charles Spaniel			Dachshund		
Genotypes	1	2	3	1	2	3
AA	1	7	2	0	0	0
AG	14	4	0	0	2	3
GG	6	0	0	1	13	0
P value	2.95×10^{-6}			1.51×10^{-3}		

Supplementary Table S4: Association between the phenotypic value and genotypes of the lead SNP in each breed as detected by a trans-breed GWAS

Breed	Phenotype	SNP	Chr	Position	MAF	Sample size	Corrected P value	Contribution (%)
Belgian Shepherd	Morphometric parameter C (height at withers)	BICF2G630361674	3	91,085,475	0.342	121	3.74.E-08	26.8
German Shepherd	Morphometric parameter C (height at withers)	TIGRP2P56799	3	91,103,945	0.235	49	4.41.E-05	35.4
Dachshund	Morphometric parameter C (height at withers)	BICF2P327350	13	31,492,811	0.146	24	1.08.E-03	42.4
Finnish Lapphund	Morphometric parameter C (height at withers)	BICF2G630662635	13	32,212,402	0.489	44	1.38.E-03	22.4
German Shepherd	Morphometric parameter C (height at withers)	BICF2P1282363	13	31,248,932	0.429	49	3.41.E-05	33.3
Labrador Retriever	Morphometric parameter C (height at withers)	BICF2S22921821	13	31,268,352	0.272	115	3.97.E-04	8.7
Dachshund	Morphometric parameter D (maximal chest diameter)	BICF2S24320436	12	61,700,493	0.396	24	5.80.E-04	41.6
Labrador Retriever	Morphometric parameter D (maximal chest diameter)	BICF2P200126	12	61,747,626	0.306	114	2.11.E-06	23.0
Dachshund	Morphometric parameter E (body length)	BICF2P274829	15	41,641,413	0.500	23	1.44.E-05	68.1
Labrador Retriever	Morphometric parameter E (body length)	BICF2P67088	15	41,206,514	0.371	115	2.34.E-04	6.8
Belgian Shepherd	Erythrocytes	BICF2G630596497	32	15,570,441	0.333	122	4.20.E-04	9.2
Finnish Lapphund	Erythrocytes	BICF2P751898	32	14,312,867	0.467	44	3.29.E-05	32.0
Belgian Shepherd	Glucose	BICF2S23023572	36	13,767,189	0.451	121	3.26.E-04	10.5
Finnish Lapphund	Glucose	BICF2P723649	36	13,192,369	0.375	45	3.35.E-06	42.5
Labrador Retriever	Glucose	BICF2P1045233	36	12,103,976	0.478	114	1.64.E-03	11.1
Cavalier King Charles Spaniel	C reactive protein	BICF2P499122	31	14,023,320	0.286	34	9.37.E-04	27.6
Newfoundland	C reactive protein	BICF2P1108776	31	15,299,470	0.452	39	3.04.E-06	48.1
Cavalier King Charles Spaniel	Heart rate during clinical exam	BICF2S2298658	7	77,826,169	0.314	35	7.87.E-04	29.6
Dachshund	Heart rate during clinical exam	BICF2P1178436	7	77,665,554	0.417	24	1.52.E-04	55.8
Cavalier king charles spaniel	Stress during physical exam	BICF2P64962	1	109,502,316	0.443	34	2.95.E-06	51.1
Dachshund	Stress during physical exam	BICF2P64962	1	109,502,316	0.146	19	1.51.E-03	44.0
Cavalier king charles spaniel	Stress during physical exam	BICF2P410245	17	12,515,438	0.186	34	4.16.E-04	31.3
Dachshund	Stress during physical exam	BICF2G630217582	17	14,737,834	0.438	19	1.91.E-07	78.4
Belgian shepherd	Stress during physical exam	BICF2G630755819	36	28,527,094	0.325	86	3.69.E-06	22.7
German shepherd	Stress during physical exam	BICF2S23444479	36	29,297,538	0.235	47	1.56.E-03	21.1

The chromosome position is based on CanFam 3.1. The p-value was corrected using the genomic control.

Supplementary Table S5: Power calcuration for P = 5 x 10^{-7} under different scenarios of effect size (0.01 – 1.5) and total sample size (50 – 10,000) with GPower 3.1

	Effect size																	
Total sample size	0.01	0.02	0.05	0.10	0.20	0.30	0.40	0.50	0.60	0.70	0.80	0.90	1.00	1.10	1.20	1.30	1.40	1.50
50	1.18.E-07	1.39.E-07	2.27.E-07	5.03.E-07	2.30.E-06	9.57.E-06	3.63.E-05	1.26.E-04	3.98.E-04	1.15.E-03	3.04.E-03	7.38.E-03	0.016	0.034	0.063	0.111	0.178	0.268
100	1.28.E-07	1.65.E-07	3.42.E-07	1.11.E-06	9.99.E-06	7.28.E-05	4.31.E-04	2.08.E-03	8.20.E-03	0.027	0.071	0.158	0.295	0.470	0.652	0.803	0.906	0.963
150	1.37.E-07	1.86.E-07	4.63.E-07	1.97.E-06	2.80.E-05	2.87.E-04	2.12.E-03	0.011	0.045	0.134	0.301	0.525	0.741	0.890	0.965	0.992	0.999	1.000
200	1.44.E-07	2.07.E-07	5.94.E-07	3.15.E-06	6.35.E-05	8.19.E-04	6.83.E-03	0.037	0.136	0.338	0.605	0.828	0.948	0.990	0.999	1.000	1.000	1.000
250	1.51.E-07	2.26.E-07	7.37.E-07	4.71.E-06	1.26.E-04	1.92.E-03	0.017	0.088	0.279	0.573	0.830	0.957	0.994	0.999	1.000	1.000	1.000	1.000
300	1.57.E-07	2.45.E-07	8.94.E-07	6.73.E-06	2.27.E-04	3.91.E-03	0.035	0.167	0.452	0.766	0.942	0.992	0.999	1.000	1.000	1.000	1.000	1.000
350	1.63.E-07	2.64.E-07	1.07.E-06	9.27.E-06	3.83.E-04	7.20.E-03	0.063	0.270	0.620	0.889	0.984	0.999	1.000	1.000	1.000	1.000	1.000	1.000
400	1.69.E-07	2.83.E-07	1.25.E-06	1.24.E-05	6.13.E-04	0.012	0.103	0.388	0.758	0.954	0.996	1.000	1.000	1.000	1.000	1.000	1.000	1.000
450	1.75.E-07	3.01.E-07	1.46.E-06	1.63.E-05	9.36.E-04	0.019	0.154	0.510	0.857	0.983	0.999	1.000	1.000	1.000	1.000	1.000	1.000	1.000
500	1.80.E-07	3.20.E-07	1.68.E-06	2.10.E-05	1.38.E-03	0.029	0.215	0.624	0.922	0.994	1.000	1.000	1.000	1.000	1.000	1.000	1.000	1.000
1,000	2.30.E-07	5.17.E-07	5.07.E-06	1.42.E-04	0.020	0.313	0.860	0.996	1.000	1.000	1.000	1.000	1.000	1.000	1.000	1.000	1.000	1.000
2,000	3.24.E-07	9.99.E-07	2.20.E-05	1.49.E-03	0.229	0.931	1.000	1.000	1.000	1.000	1.000	1.000	1.000	1.000	1.000	1.000	1.000	1.000
3,000	4.19.E-07	1.64.E-06	6.33.E-05	6.81.E-03	0.605	0.999	1.000	1.000	1.000	1.000	1.000	1.000	1.000	1.000	1.000	1.000	1.000	1.000
5,000	6.29.E-07	3.51.E-06	2.97.E-04	0.048	0.969	1.000	1.000	1.000	1.000	1.000	1.000	1.000	1.000	1.000	1.000	1.000	1.000	1.000
10,000	1.30.E-06	1.33.E-05	3.46.E-03	0.420	1.000	1.000	1.000	1.000	1.000	1.000	1.000	1.000	1.000	1.000	1.000	1.000	1.000	1.000

Blue boxes show statistical power > 0.8.

Supplementary Table S6: Dog group information

Breeds		Country		Sex	Number of dogs
Belgian Shepherd	BS	Belgium	BE	Male	95
		France	FR	Male	28
Cavalier King Charles Spaniel	CK	Sweden	SW	Male	35
Dachshund	DH	Finland	FI	Male	24
Doberman	DM	France	FR	Male	38
Finnish Lapphund	FL	Finland	FI	Male	45
German Shepherd	GS	Finland	FI	Male	49
Labrador Retriever	LR	Denmark	DK	Female	43
		France	FR	Female	28
		Sweden	SW	Male	45
Newfoundland	NF	Denmark	DK	Female	42

Supplementary Figure S1: Schema for analyses in this study

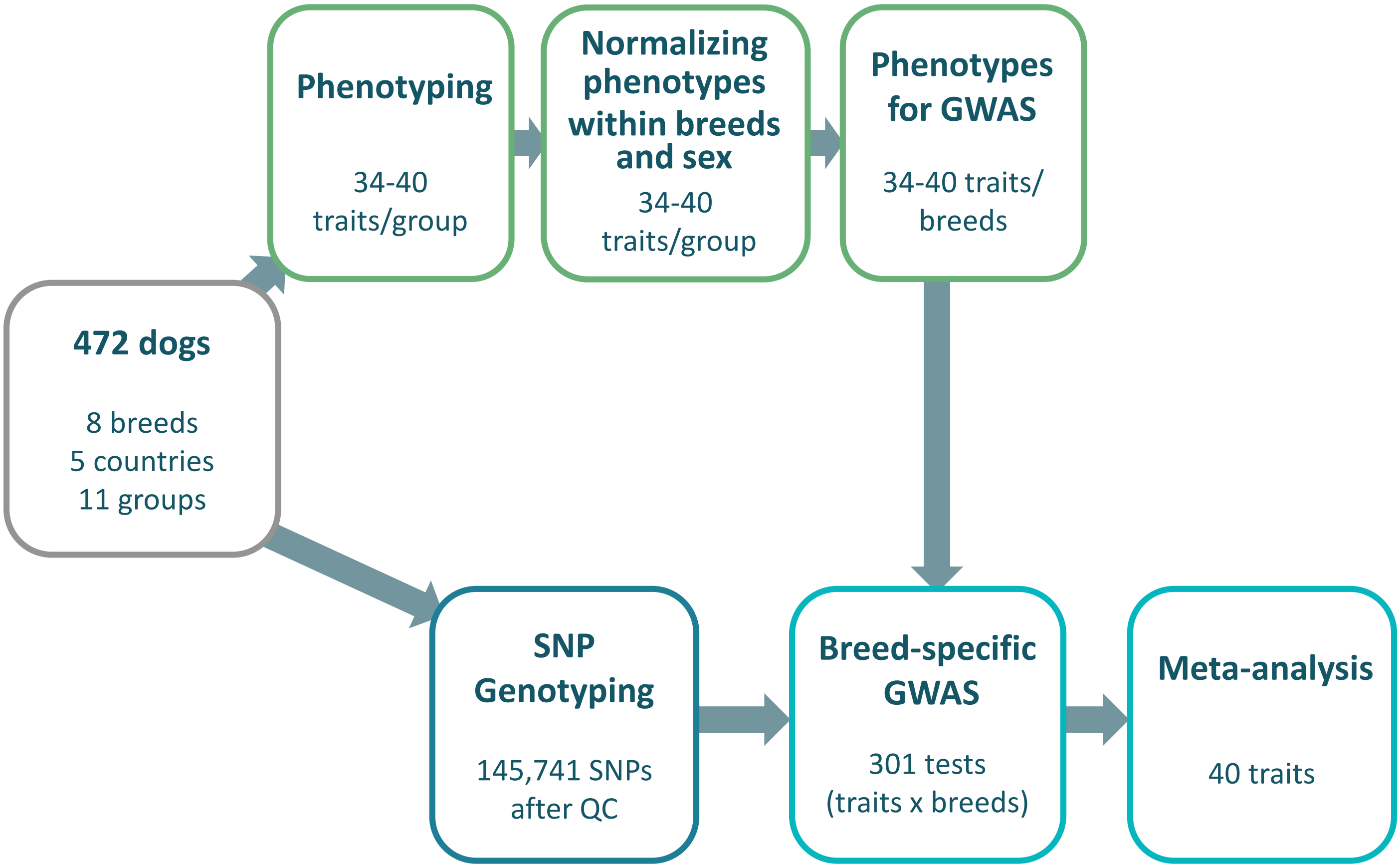

Supplementary Figure S2: The phenotype distribution for each genotype at the most associated SNPs detected by a breed-specific GWAS

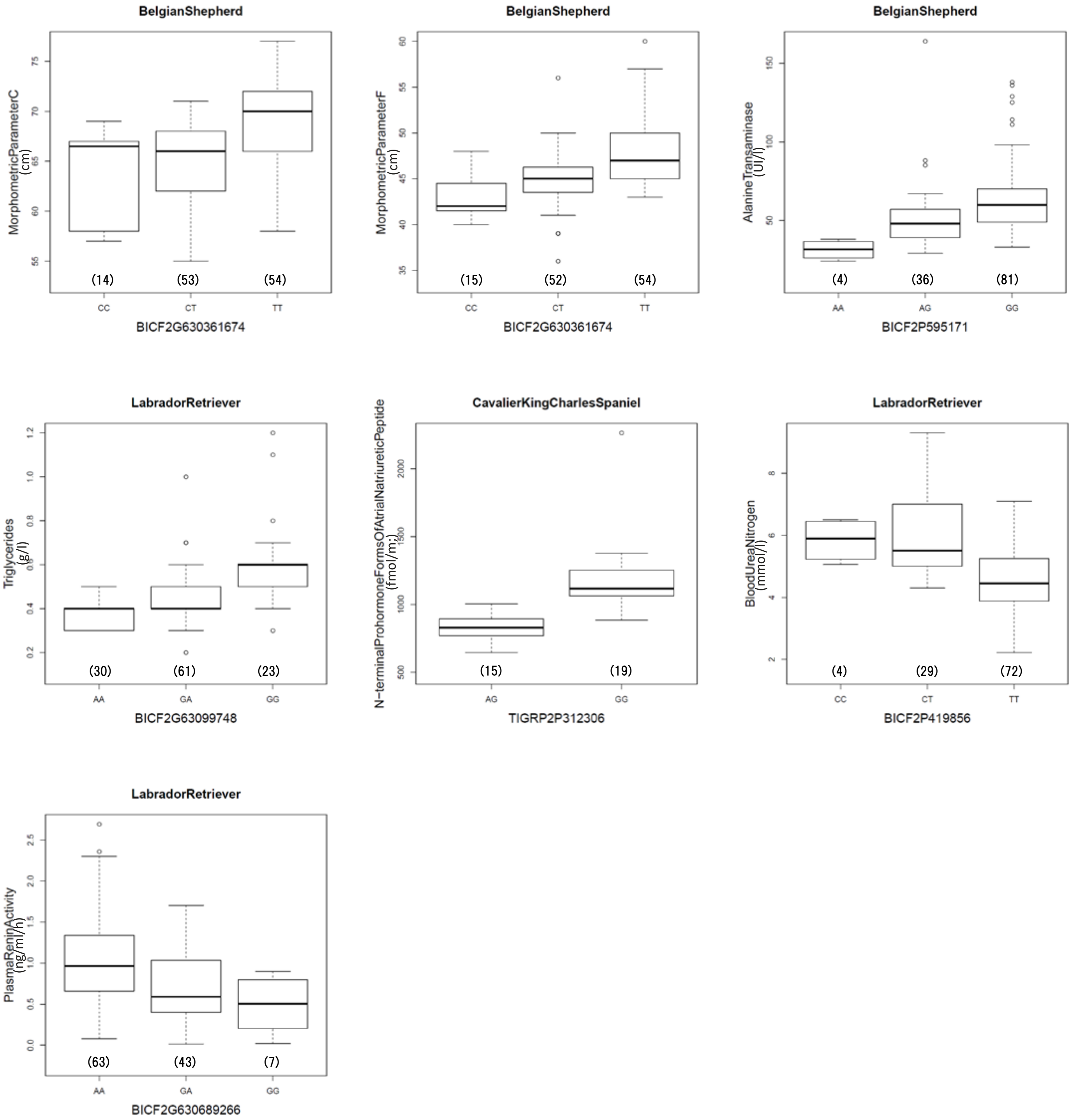

Supplementary Figure S3: QQ plots for ALT and fructosamine

a

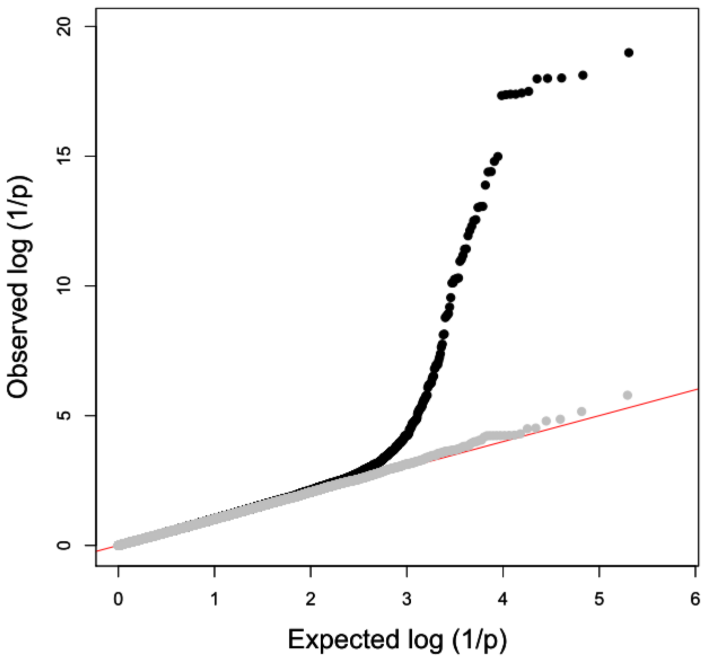

b

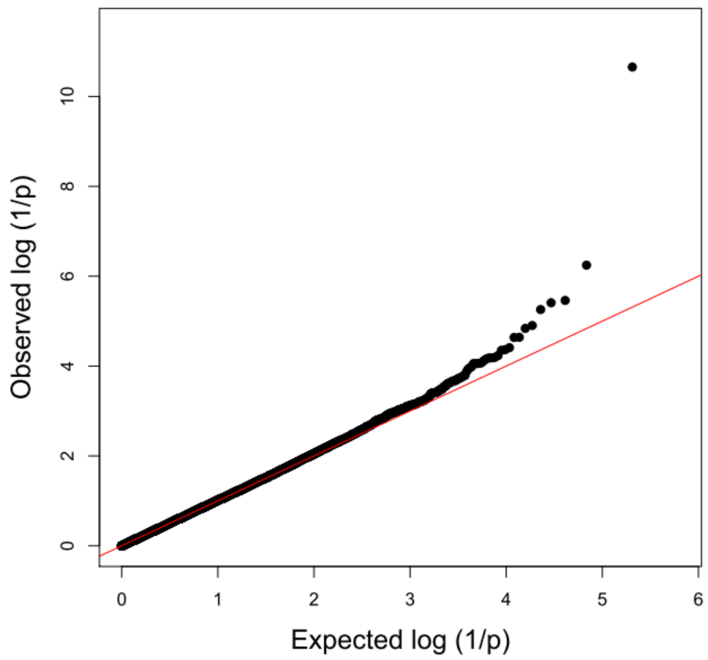

(a) QQ plot for alanine aminotransferase (ALT). Black spots indicate all SNPs and grey spots indicate SNPs on chromosomes other than 13 (b) QQ plot for fructosamine

Supplementary Figure S4: Linkage disequilibrium map between 36 MBp and 40 MBp on chromosome 13

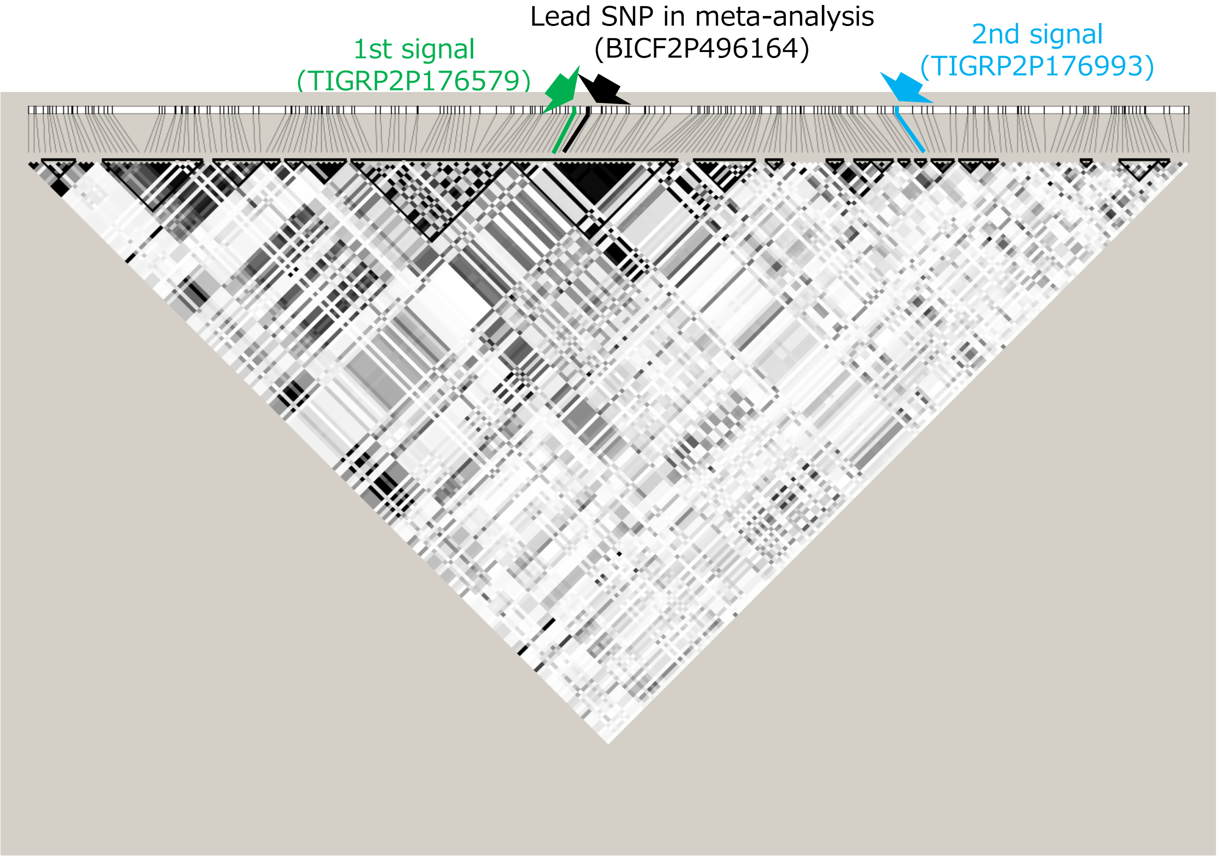

Supplementary Figure S5: Morphometric parameters A-G measured in this study

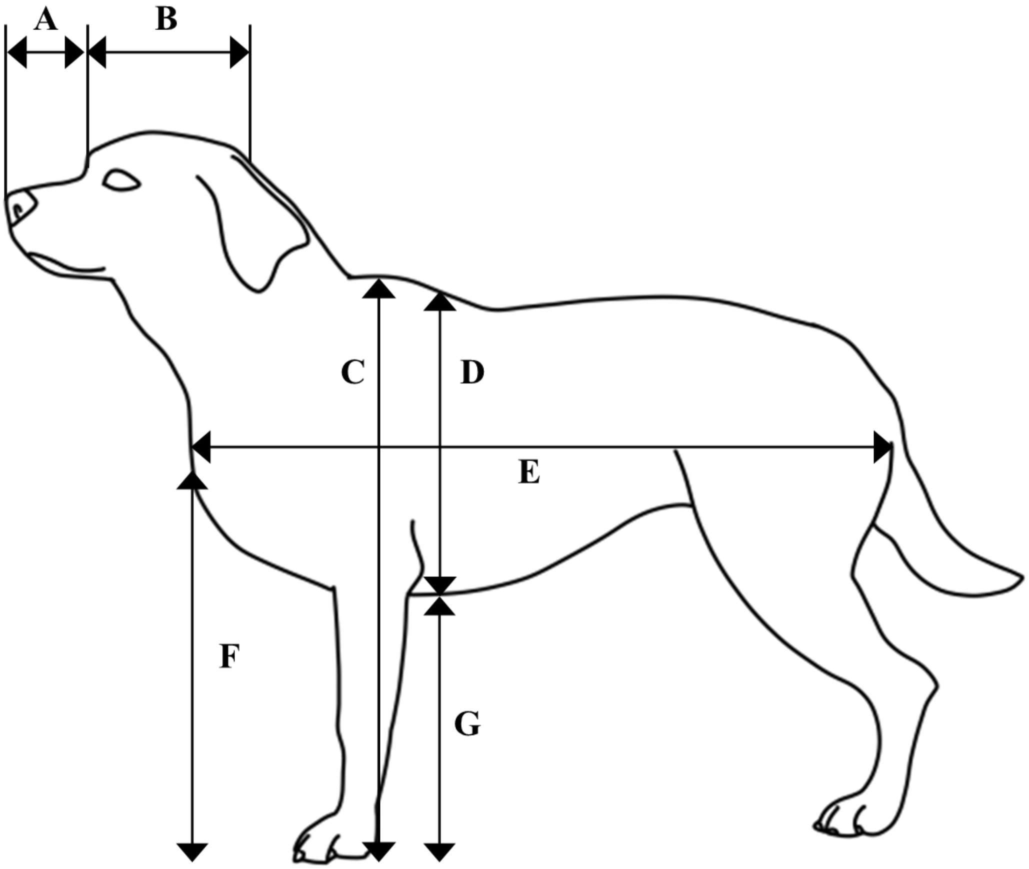

Morphometric parameters	Description
A	Snout length
B	Head length
C	Height at withers
D	Maximal chest diameter
E	Body length
F	Height at point of shoulder
G	Height at olecranon

This dog was drawn by Ms. Misaki Mizukoshi (Laboratory for Genotyping Development, RIKEN Center for Integrative Medical Sciences, Japan)

Supplementary Figure S6: Association between the values for morphometric parameters C (height at withers) and E (body length) and the dog breeds

a

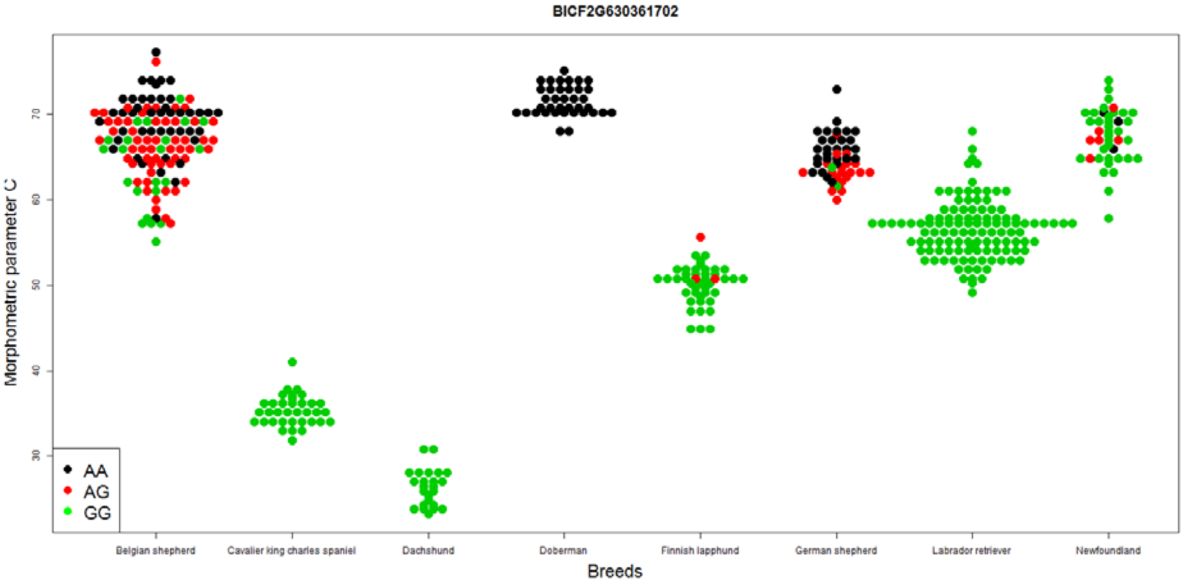

b

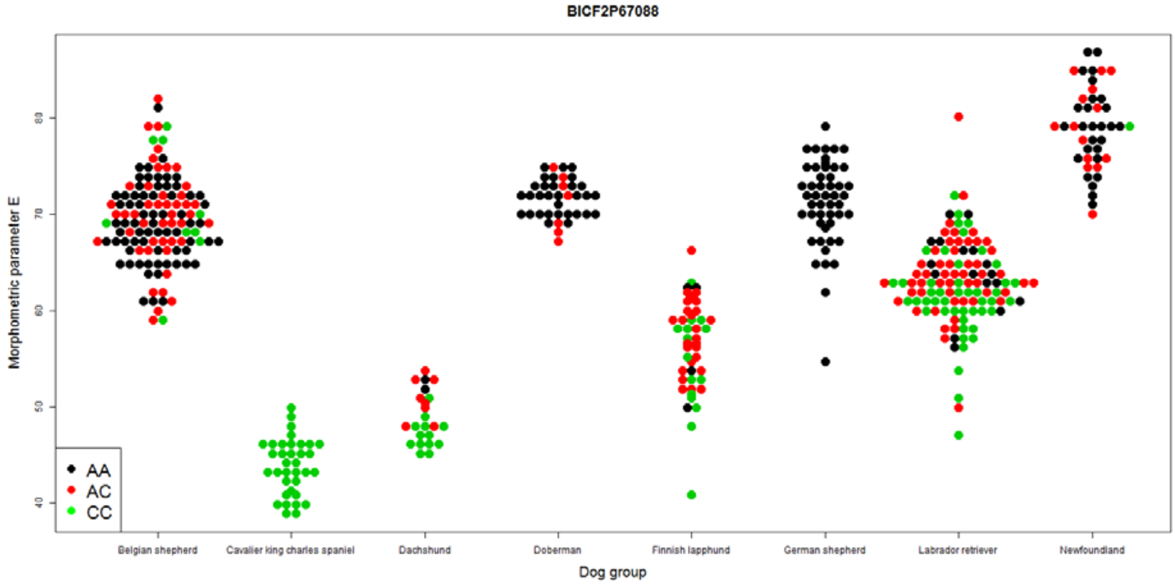

Supplementary Figure S7: The phenotype distribution for each genotype at the most associated SNPs as detected by a meta-analysis

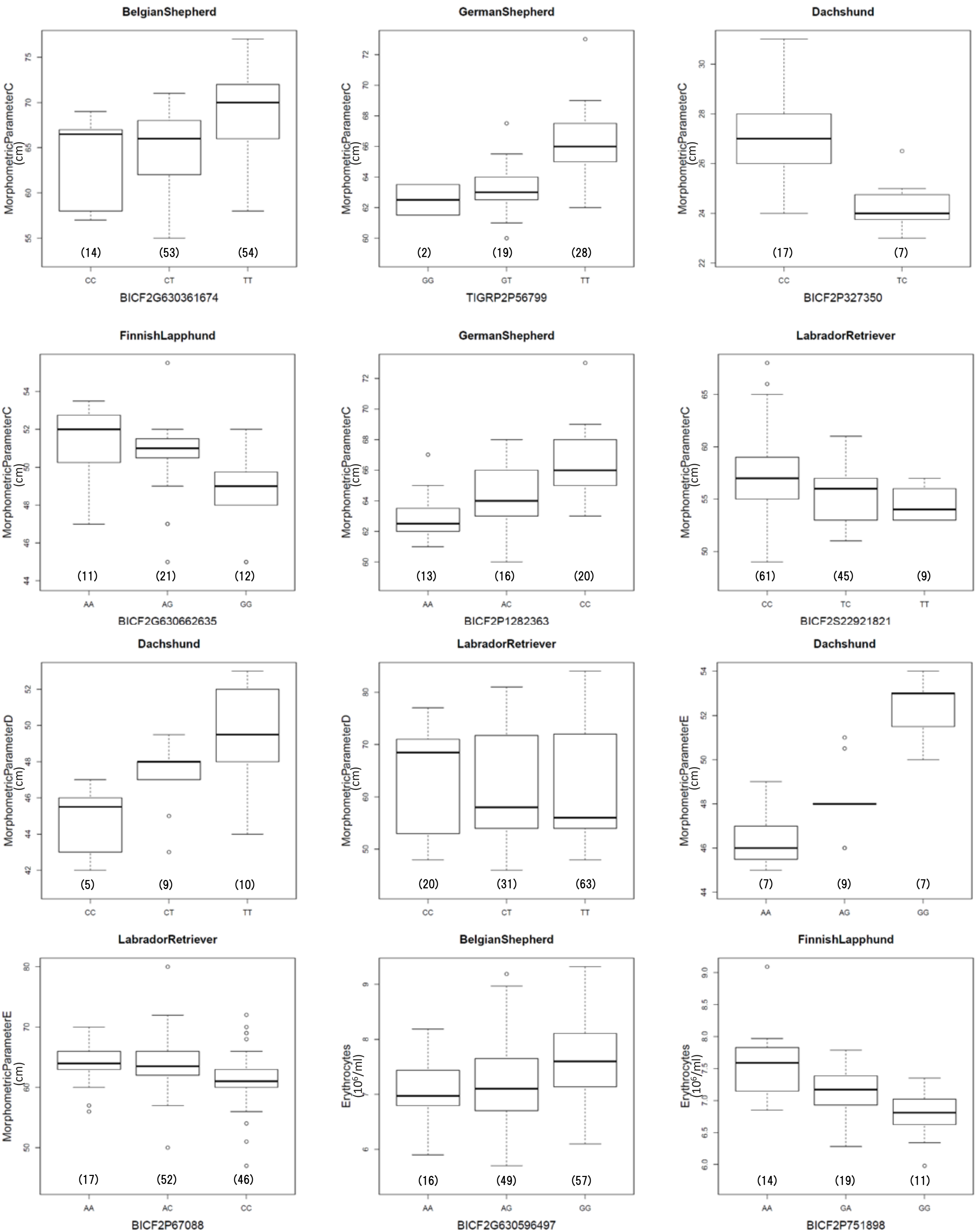

Supplementary Figure S7 (continued)

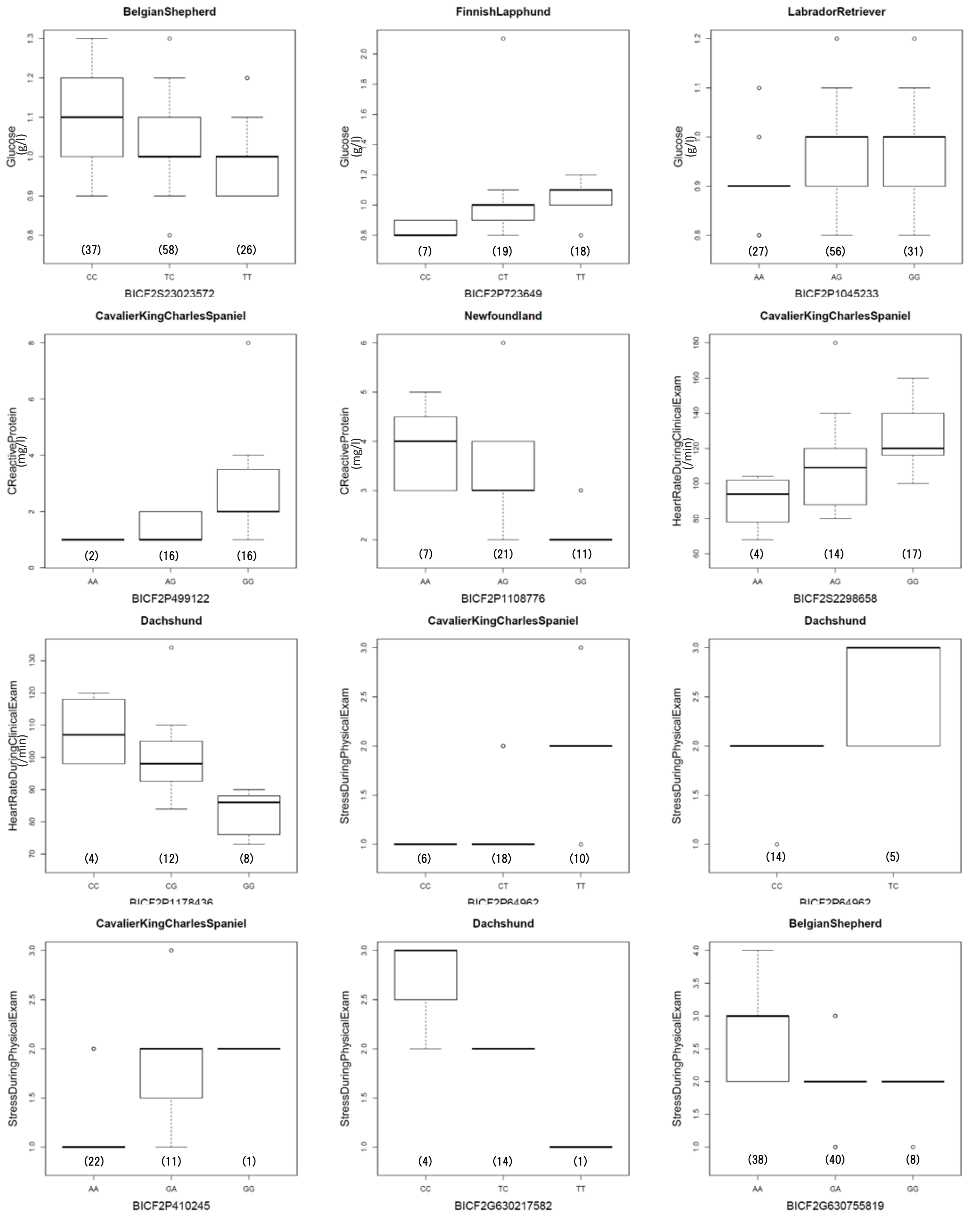

Supplementary Figure S7 (continued)

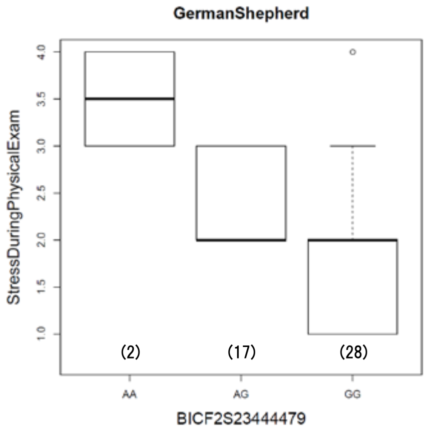

Supplementary Figure S8: Phenotype difference between countries in female Labrador Retriever

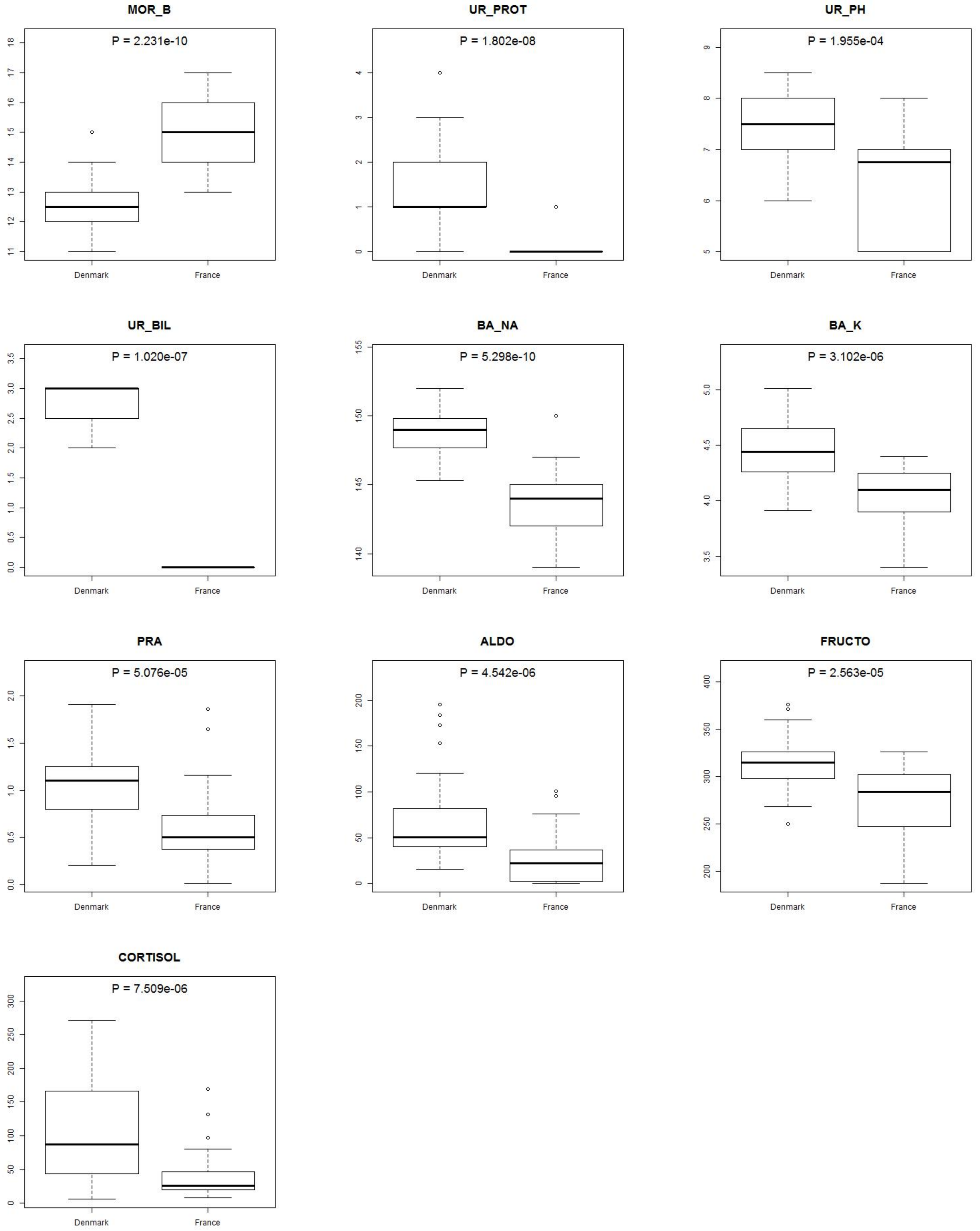

Supplementary Figure S9: Phenotype difference between countries in male Belgian Shepherd

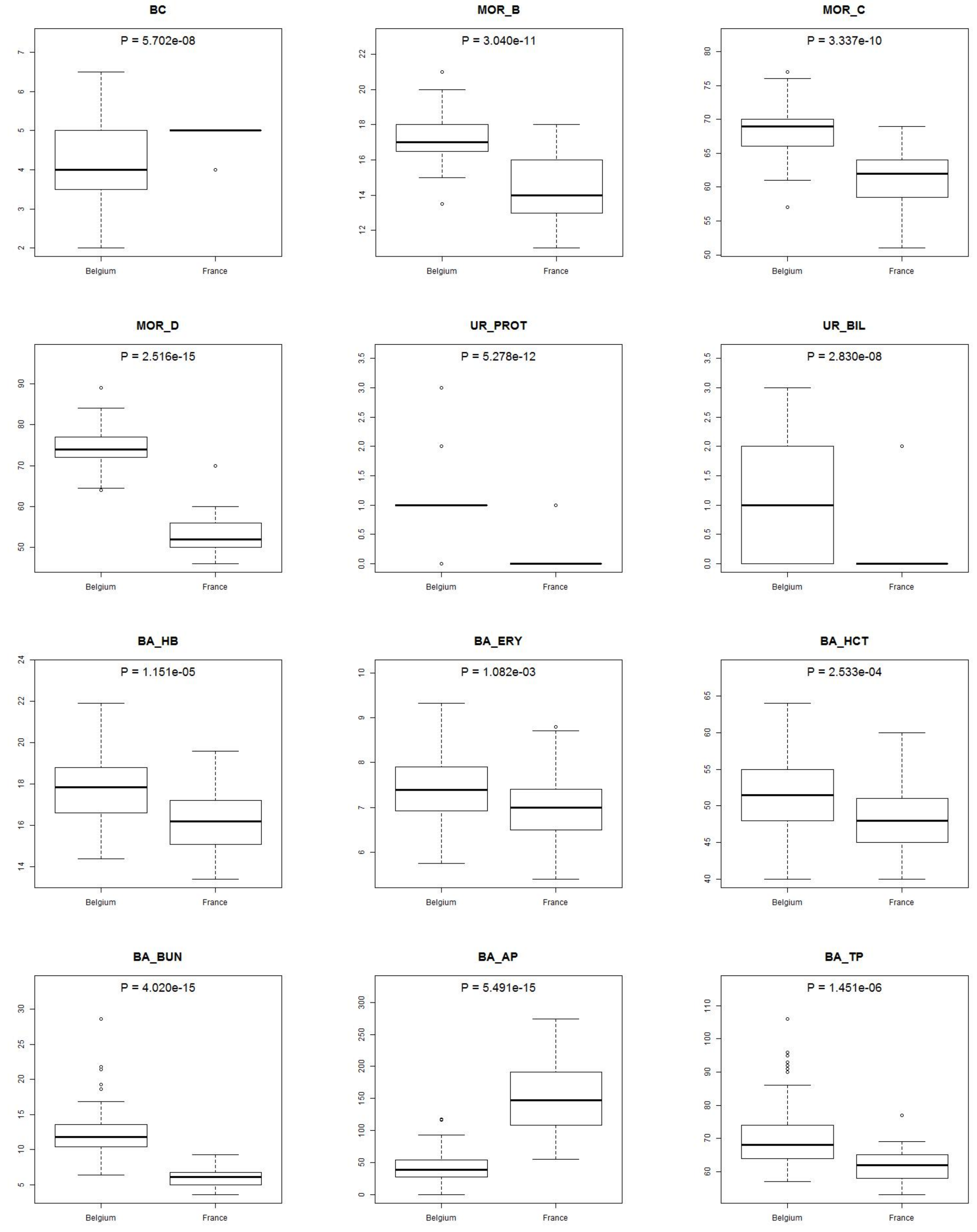

Supplementary Figure S9 (continued)

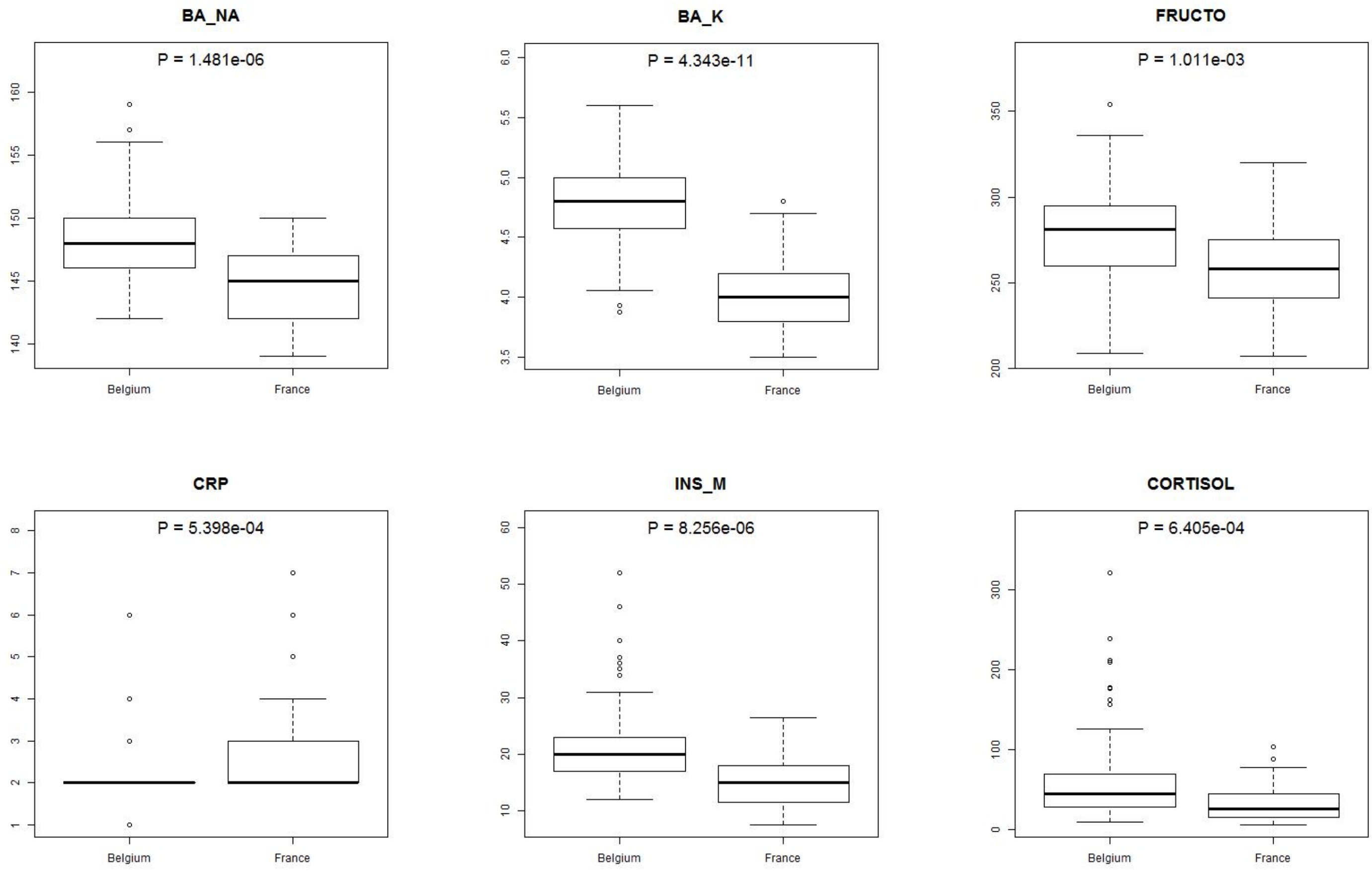

Supplementary Figure S10: Results of the principle component analysis

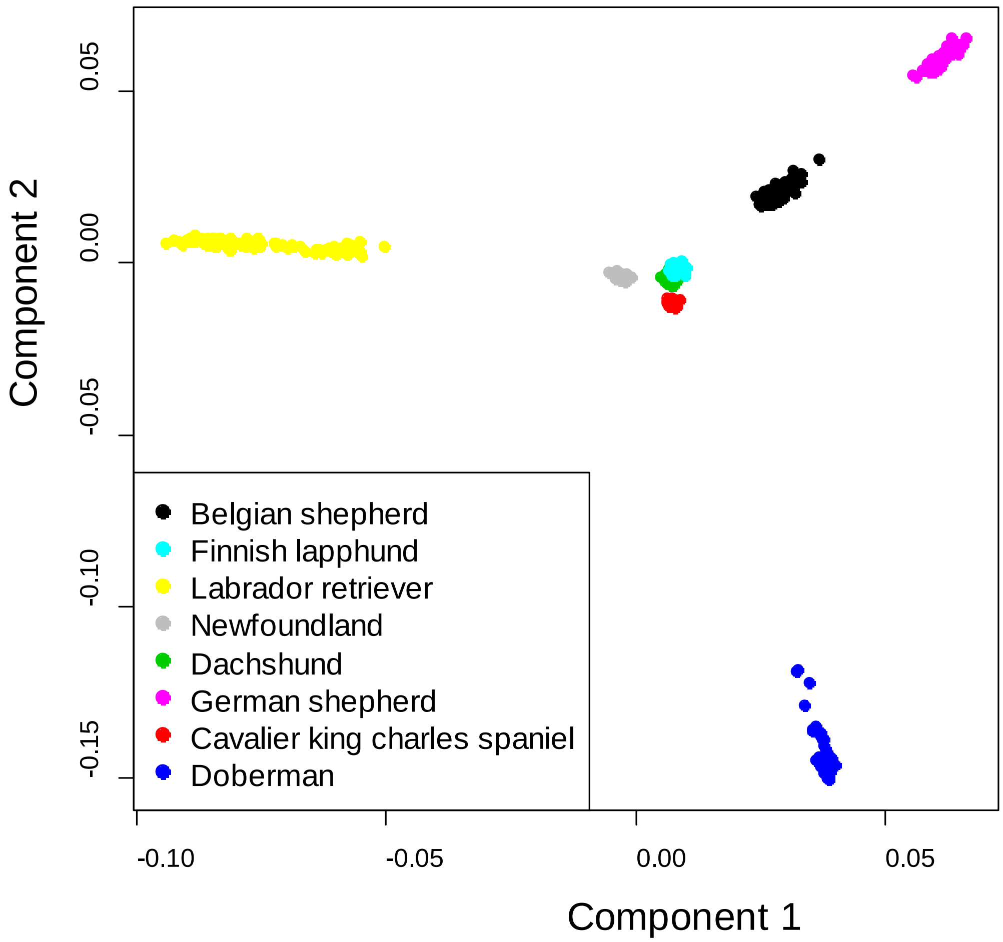

Supplement: Supplementary file 1 — Supplementary information. [file 41598_2020_63457_MOESM1_ESM.pdf]
